# Microbial Syntrophy Drives Methanogenesis From Residual Hydrocarbons in Anaerobic Oil Sands Tailings

**DOI:** 10.1111/1462-2920.70470

**Published:** 2026-11-02

**Authors:** Ali Hamidoğlu, Hao Wang

**Affiliations:** ^1^ Research Center for Applied and Computational Mathematics Khazar University Baku Azerbaijan; ^2^ Interdisciplinary Lab for Mathematical Ecology and Epidemiology (ILMEE) & Department of Mathematical and Statistical Sciences University of Alberta Edmonton Alberta Canada

**Keywords:** anaerobic biodegradation, bacterial–archaeal interactions, microbial processes, oil sands tailings, petroleum hydrocarbons, Shapley analysis

## Abstract

Anaerobic oil sands tailings can generate methane for decades as residual naphtha‐range and petroleum hydrocarbons sustain microbial metabolism, but how microbial interactions link hydrocarbon turnover to methanogenesis remains poorly resolved. To characterize these microbial associations, we analyse two naphtha‐amended anaerobic tailings incubation series using 16S rRNA gene relative abundance, hydrocarbon consumption, and methane production data within a cooperative game‐theoretical framework. The abundance‐informed Shapley analysis attributes system‐level variation to bacterial–archaeal coalitions, highlighting key syntrophic partnerships. Several taxa show inferred contributions to methanogenesis that are disproportionate to their relative abundance, indicating that model‐based attribution can differ from abundance ranking. Sensitivity analysis shows that the main methane‐attribution patterns remain interpretable across alternative abundance‐weighted coefficient formulations. These interaction patterns shift across incubation phases, showing that microbial control over hydrocarbon‐dependent methanogenesis changes through time. When incorporated into stochastic forecasting, Shapley‐derived reaction intensities improve predictive performance compared with reduced microbial and non‐microbial benchmarks. Together, our results indicate that methane production in oil sands tailings can be interpreted through abundance‐informed bacterial–archaeal coalition structure, providing a coalition‐aware basis for interpreting and forecasting methanogenic hydrocarbon transformation.

## Graphical abstract

Methane production from residual hydrocarbons in anaerobic oil sands tailings is shaped by interactions between bacterial and archaeal groups. Shapley‐based attribution reveals differences between microbial contributions and relative abundance, while variation across sites and stages highlights the role of environmental context in methane production and late‐stage prediction.

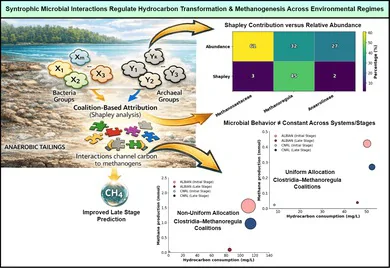

## 1 Introduction

Anaerobic oil sands fine tailings represent a large water‐saturated, industrial residual system that retains residual naphtha‐range diluent and other petroleum hydrocarbons that undergo slow methanogenic transformation and sustain long‐term methane (CH_4_) generation (Hamidoğlu and Wang 2026; You et al. 2020). Under these anoxic conditions, hydrocarbon turnover depends on sequential bacterial and archaeal interactions that transfer carbon and electrons through the anaerobic food web until methanogenic archaea complete the process (Xu et al. 2024). Methanogenic conversion of short‐chain n‐alkanes and BTEX‐type compounds from naphtha has been demonstrated using tailings‐derived enrichment cultures, showing that hydrocarbon consumption and methane formation are tightly linked to community dynamics and biogeochemical transformations rather than a single organism (Siddique et al. 2012). Long incubations with naphtha‐amended mature fine tailings (MFT) show that hydrocarbons are consumed sequentially over years, after long lag phases, while bacterial and archaeal communities shift as different compounds disappear and new intermediates appear (Siddique et al. 2015). Tailings from different sites can show different lag times, different preferred hydrocarbons, and different dominant coalitions during active degradation (Shahimin et al. 2016). Together, these studies show that hydrocarbon consumption and methane release in anaerobic tailings depend on microbial interactions that vary across sites and over time (An et al. 2013). Magnetite has been reported to enhance hydrocarbon biodegradation and CH_4_ production in oil sands tailings by promoting microbial cooperation and electron‐transfer processes (Afzal, Kuznetsova, et al. 2025). This shows that methane‐producing hydrocarbon degradation can be influenced by environmental factors that reshape microbial interactions. In addition, anaerobic petroleum‐hydrocarbon biodegradation under methanogenic conditions depends on syntrophic organization, with Syntrophaceae and their partnerships playing a quantitatively important role in alkane turnover (Gray et al. 2011).

Despite this progress, many quantitative analyses in Environmental Microbiology still rely on correlation analysis, co‐occurrence networks, or differential abundance patterns to interpret community shifts, even though these approaches primarily describe community structure rather than microbial connectivity or functional exchange (Oña et al. 2025; Friedline et al. 2025; Li et al. 2023; De Vrieze and Verstraete 2016). These approaches identify microbial associations, but they do not quantify each group's contribution to methane production. They cannot distinguish direct metabolic hand‐offs from indirect responses to shared environmental drivers, and key metabolic roles are often carried by poorly resolved or uncultivated partners whose importance is not captured by abundance patterns alone (Narihiro et al. 2015). As a result, current tools provide limited guidance on which microbial groups actually carry methane‐forming carbon through the anaerobic network and how those roles reorganize across ecological phases.

Long‐term incubation studies of oil sands tailings show that methanogenic hydrocarbon biodegradation proceeds through staged changes in hydrocarbon consumption, methane production, and microbial community structure (Shahimin and Siddique 2017; Siddique et al. 2015, 2020). These staged changes support the view that methane formation in tailings emerges from dynamic microbial interactions rather than from the most abundant taxa alone (Gray et al. 2011; Harindintwali et al. 2023). Common community profiling approaches can describe which taxa are present or which genes are detected, but they remain limited for direct functional inference from taxonomic data alone (Matchado et al. 2024; Djemiel et al. 2022). This creates a practical attribution problem in cooperative microbial systems because these approaches do not by themselves quantify the marginal contribution of a taxon when functions are shared across guilds or when outcomes depend on microbial cooperation.

Post hoc feature‐attribution methods can be useful for interpreting predictive models, but their rankings can be sensitive to preprocessing, correlated features, feature representation, and model structure (Zhou, Booth, et al. 2022; Verdinelli and Wasserman 2024; Hwang et al. 2025; Huang and Marques‐Silva 2024; Karwowska et al. 2025). In microbiome applications, ranked taxa should therefore be interpreted as model‐dependent indicators rather than direct evidence of microbial function (Novielli et al. 2024; Przymus et al. 2025; Dai et al. 2024). For methanogenic hydrocarbon degradation, this distinction is especially important because hydrocarbon activation, syntrophic transfer, and terminal methanogenesis can be distributed across different bacterial and archaeal groups (Siddique et al. 2012; Xu et al. 2024; Zhou, Zhang, et al. 2022; Huang et al. 2025). These limitations highlight the need for an attribution framework that is defined directly on observed biogeochemical outcomes and can explicitly evaluate microbial coalitions.

This conceptual gap motivates the use of cooperative game theory in the present study. Rather than explaining a black‐box predictor, we define the game directly on observed methane generation and hydrocarbon depletion trajectories in anaerobic oil sands tailings incubations under tailings‐relevant geochemical regimes. Bacterial and archaeal guilds are treated as players, and the coupled biogeochemical response constitutes the value function. In this formulation, Shapley values are coalition‐aware allocations that quantify how each microbial group contributes in the presence of all possible partners, thereby capturing synergy, redundancy, and syntrophic hand‐offs that are invisible to standard association‐based analyses. By embedding these allocations within a stochastic, regime‐based forecasting framework, we test how microbial interactions shape coupled methane–hydrocarbon dynamics through time and identify the coalitions most relevant to long‐term forecasting and mitigation in anaerobic tailings systems. To our knowledge, this is the first study to analyse anaerobic oil sands tailings microbiology using an explicit cooperative game‐theoretic framework, enabling coalition‐aware quantification of bacterial–archaeal contributions to methane‐forming carbon transfer beyond traditional correlation‐based analyses.

## 2 Methods

This section is organized as follows: Section 2.1 describes the datasets employed, including site‐specific tailings compositions, incubation measurements, and microbial community profiles, and details preprocessing steps to ensure reproducibility. Section 2.2 presents a novel cooperative game formulation tailored to anaerobic oil sands tailings, with regime‐specific Shapley value calculations to quantify phase‐dependent microbial contributions during incubation. Section 2.3 integrates the Shapley values with time‐series modelling using L1 regularization, called the least absolute shrinkage and selection operator (Lasso) (Tibshirani 1996), and stochastic Monte Carlo simulations, enabling interpretable forecasting of methane–hydrocarbon dynamics over long‐term incubations. Finally, Section 2.4 provides the overall workflow of the study.

### 2.1 Data Description and Implementation

This section outlines the sources of experimental data and the preprocessing methods required for the preparation of numerical results. As a case study, we investigate naphtha‐amended anaerobic microbial communities reported by Shahimin and Siddique (2017) for MFTs collected from the Canadian Natural Resources Limited (CNRL) and ALBIAN oil sands operations. The labels ALBIAN and CNRL are used here only as site/sample identifiers reported in the source study; no company involvement or endorsement is implied in the present analysis. These datasets provide a well‐characterized benchmark for evaluating Shapley attributions of microbial interactions under long‐term methanogenic conditions.

The microbial composition and 16S rRNA gene relative abundance data are presented in Table 1, showing differences in community structure between the two sites. Because absolute microbial loads, biomass estimates, or qPCR‐normalized abundances were not available in the source dataset, these differences are interpreted as changes in relative community composition rather than absolute microbial abundance.

**TABLE 1:** The 16S rRNA gene relative abundance (%) of dominant bacterial and archaeal taxa in naphtha‐amended ALBIAN and CNRL MFTs at selected incubation times.

Taxonomic group	ALBIAN MFT (%)			CNRL MFT (%)		
	Day 0	Day 400	Day 900	Day 0	Day 400	Day 900
Bacteria						
Betaproteobacteria	90.3	3.2	0.5	—	—	—
Actinobacteria	—	—	—	19.7	7.5	1.2
Anaerolineae	1.4	27.0	8.6	23.9	31.1	2.0
Clostridia	0.1	38.8	78.5	1.9	0.1	65.2
Deltaproteobacteria	—	—	—	5.8	24.6	19.4
Others (< 5%)	8.2	31.0	12.4	48.7	36.7	12.2
Archaea						
Methanosaetaceae	63.1	60.8	28.1	60.2	80.6	8.1
Methanosarcinaceae	15.5	2.3	1.4	—	—	—
Methanoregula	2.6	32.3	66.2	26.1	17.3	91.3
Others (< 5%)	18.8	4.6	4.3	13.7	2.1	0.6

*Note:* “Others” represents the sum of taxa individually contributing less than 5% of total reads.

In ALBIAN MFTs, the bacterial community undergoes a clear transition from early dominance by Betaproteobacteria, primarily Burkholderiales, Comamonadaceae, Hydrogenophilales, Hydrogenophilaceae, and Thiobacillus at day 0, toward a Clostridia‐dominated assemblage by day 900, including Clostridiales, Peptococcaceae, and Cryptanaerobacter, with a sustained but moderate presence of Anaerolineae (Anaerolineales, Anaerolineaceae, and Leptolinea).

In contrast, CNRL MFTs exhibit a more heterogeneous bacterial structure during early incubation, characterized by substantial contributions from Anaerolineae, Actinobacteria (notably Coriobacteriales and Coriobacteriaceae), and Deltaproteobacteria, including key syntrophic taxa such as Geobacter, Smithella, and Syntrophus. This is followed by a rapid enrichment of Clostridia by day 900, indicative of faster syntrophic network establishment and accelerated hydrocarbon turnover.

Archaeal succession further reinforces this divergence: ALBIAN MFTs display a gradual replacement of acetoclastic Methanosaetaceae by hydrogenotrophic Methanoregula, whereas CNRL MFTs undergo a sharp late‐stage dominance of Methanoregula, exceeding 90% by day 900, signalling a strong shift toward hydrogenotrophic methanogenesis. In our setting, we consider Betaproteobacteria, Anaerolineae, Clostridia and others as 4 different bacteria groups $\left(m=4\right)$ for ALBIAN case, while we investigate Actinobacteria, Anaerolineae, Clostridia, Deltaproteobacteria, and others as 5 different bacteria groups $\left(m=5\right)$ for CNRL case. The ALBIAN case categorizes Methanosaetaceae, Methanosarcinaceae, Methanoregula, and others into four distinct archaeal groups $\left(k=4\right)$, whereas the CNRL case combines Methanosaetaceae, Methanoregula, and others into three distinct archaeal groups $\left(k=3\right)$ within our study.

Methanogenic conditions are shown in Table 2 for hydrocarbon consumption and cumulative methane production in live ALBIAN and CNRL MFT microcosms. Because intermediate hydrocarbon concentrations are not tabulated in the source study, values at 400, 900, and 1300 days are reconstructed using monotonic linear interpolation between the nearest experimentally measured time points. Missing methane values at 400, 600, 900, and 1300 days are obtained from the cumulative methane production curves using the Plotdigitizer software. The measured methane values at 0 and 1600 days are retained as reported, and all interpolated points are clearly marked in Table 2.

**TABLE 2:** Hydrocarbon consumption and cumulative methane production in naphtha‐amended live ALBIAN and CNRL MFT microcosms are presented under methanogenic conditions.

Time (d)	ALBIAN HC (mg/L)	CNRL HC (mg/L)	ALBIAN CH_4_ (mmol/100 mL)	CNRL CH_4_ (mmol/100 mL)
0	1833^a^	2351^a^	0.00^a^	0.00^a^
400	1533^b^	2044^b^	1.83^c^	0.76^c^
600	1383^a^	1891^a^	1.88^c^	1.79^c^
900	1229^b^	1793^b^	2.09^c^	1.8^c^
1300	1042^b^	1663^a^	2.2^c^	2.07^c^
1600	870^a^	1292^a^	2.38^a^	3.00^a^

*Note:* Experimentally reported values are taken directly from the source; for missing hydrocarbon data, they are evaluated by using the linear interpolation technique, and for missing methane data, the Plotdigitizer is used as software to extract data from the source.

^a^ Experimentally measured data.

^b^ Hydrocarbon concentrations estimated via monotonic linear interpolation between experimentally reported sampling points.

^c^ Methane values derived using the Plotdigitizer software on reported cumulative methane production curves.

### 2.2 Cooperative Game Formulation

This section presents a Shapley game perspective for microbial communities in anaerobic methane‐producing environments, focusing on cooperative interactions among bacterial and archaeal groups. Here, we reinterpret classical cooperative game theory concepts within a microbial ecological framework. In economic terminology, a player is an agent that contributes to a joint outcome. In this study, a player represents a microbial taxonomic group used in the model, not an individual DNA sequence, amplicon sequence variant (ASV), or operational taxonomic unit (OTU). Thus, m bacterial groups, denoted $X_{1},X_{2},\ldots ,X_{m}$, and k archaeal groups, denoted $Y_{1},Y_{2},\ldots ,Y_{k}$, are treated as players. Each player represents a group‐level microbial category with a possible ecological role in hydrocarbon transformation, fermentation, or methanogenesis. These roles are interpreted at a broad guild level and are not direct functional assignments to individual ASVs or OTUs.

A coalition refers to a subset of players that cooperate to generate value. Biologically, coalitions represent possible syntrophic consortia, where bacterial and archaeal groups interact through linked metabolic processes. In the classical syntrophic interpretation of anaerobic hydrocarbon biodegradation, bacterial groups contribute to hydrocarbon transformation and fermentation, producing intermediates such as acetate and hydrogen, while methanogenic archaea consume downstream products and generate methane. In this framework, methane production is attributed to bacteria–archaea coalitions that link hydrocarbon transformation to methanogenesis. Thus, coalitions represent model‐implied microbial cooperation rather than simple taxonomic co‐occurrence or direct ASV‐level functional prediction.

A payoff represents the value generated by a coalition. In our system, the payoff is defined as a two‐dimensional biogeochemical output: methane production (M units) and hydrocarbon consumption (C units). Here, C denotes the observed hydrocarbon consumption over a given incubation regime, not the initial hydrocarbon input. Thus, hydrocarbon consumption is treated as a measured degradation outcome, while methane production is treated as a downstream methanogenic outcome. Under this formulation, the grand coalition, which contains all bacterial and archaeal groups, is assigned the total observed outcome $\left(M,C\right)$. The Shapley value then distributes these two system‐level outcomes back to individual microbial groups according to their marginal contributions across possible bacteria–archaea coalition structures. In ecological terms, the framework identifies microbial groups with high model‐implied contributions to hydrocarbon consumption and methane production, rather than simply identifying the most abundant taxa. Based on this methodology, we define the two‐dimensional game value as follows:
(1)

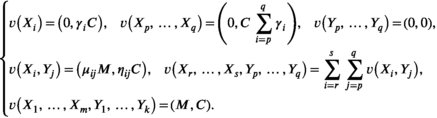

here $v\left(S\right)=\left(v_{M}\left(S\right),v_{C}\left(S\right)\right)$ is a vector‐valued payoff, where $v_{M}\left(S\right)$ denotes the methane‐production component and $v_{C}\left(S\right)$ denotes the hydrocarbon‐consumption component of coalition S. Thus, methane production and hydrocarbon consumption are not combined into a single scalar quantity. The Shapley allocation is applied componentwise, so methane and hydrocarbon contributions are interpreted separately. The coefficient $\gamma_{i}>0$ represents the individual bacterial hydrocarbon‐consumption weight, while $\mu_{\mathrm{ij}}>0$ and $\eta_{\mathrm{ij}}>0$ represent bacteria–archaea interaction effects on methane production and hydrocarbon consumption, respectively, with $i\in \left\{1,\ldots ,m\right\}$ and $j\in \left\{1,\ldots ,k\right\}$. These weights satisfy

(2) $$\sum_{i=1}^{m}\sum_{j=1}^{k}\mu_{\mathrm{ij}}=1,\;\text{and}\;\sum_{i=1}^{m}\sum_{j=1}^{k}\eta_{\mathrm{ij}}=1.$$

The methane and hydrocarbon components of the value function are used for attribution, not for carbon mass balance. Therefore, the model does not assume that consumed hydrocarbon carbon is converted one‐to‐one into methane. Carbon may also be directed to biomass, fermentation intermediates, dissolved inorganic carbon, or other unmeasured sinks. The methane and hydrocarbon components are therefore interpreted as coupled but separate observed outcomes of the incubation. The weights $\mu_{\mathrm{ij}}$ and $\eta_{\mathrm{ij}}$ parameterize the contribution of each bacteria–archaea interaction to the methane‐production and hydrocarbon‐consumption components of the value function, respectively. The Shapley value then uses this value function to allocate the observed methane and hydrocarbon outcomes among microbial groups. Because $\mu_{\mathrm{ij}}$ and $\eta_{\mathrm{ij}}$ are normalized separately, the framework does not impose perfect stoichiometric coupling between hydrocarbon consumption and methane production.

Since the game value function is componentwise increasing with coalition size, the game satisfies the convexity condition stated in Definition A.1 in Appendix A (see also Theorem B.1 in Appendix B). Consequently, the core is non‐empty and the Shapley value lies within, yielding the equilibrium in distribution that is both fair and stable (see Theorem B.3 in Appendix B). Here, we find the corresponding Shapley values as follows:(see Theorem B.2 in Appendix B):

(3) $$\phi_{X_{i}}≔\left(\phi_{M}\left(X_{i}\right),\phi_{H}\left(X_{i}\right)\right)=\left(\frac{M}{2}\sum_{\ell =1}^{k}\mu_{i\ell },\frac{C}{2}\sum_{\ell =1}^{k}\eta_{i\ell }+C\frac{\gamma_{i}}{k+1}\right),$$

(4) $$\phi_{Y_{j}}≔\left(\phi_{M}\left(Y_{j}\right),\phi_{H}\left(Y_{j}\right)\right)=\left(\frac{M}{2}\sum_{\ell =1}^{m}\mu_{\ell j},\frac{C}{2}\sum_{\ell =1}^{m}\eta_{\ell j}-C\frac{\sum_{i=1}^{m}\gamma_{i}}{k\left(k+1\right)}\right),$$

where $i\in \left\{1,2,\cdots ,m\right\}$ and $j\in \left\{1,2,\cdots ,k\right\}$.

The Shapley values are first computed at the microbial‐group level given in Equations (3) and (4) and then redistributed to bacteria–archaea pairs. In this regard, we use two decomposition strategies: a uniform (equal‐sharing) and an interaction‐weighted (non‐uniform) allocation. Both preserve total methane production and hydrocarbon consumption and differ only in how group‐level Shapley values are assigned to pairs.

Under the uniform allocation strategy, for each pairwise coalition $\left(X_{i},Y_{j}\right)$, the methane and hydrocarbon Shapley contributions, denoted by $\phi_{M}\left(X_{i},Y_{j}\right)$ and $\phi_{H}\left(X_{i},Y_{j}\right)$, respectively, are defined as follows:

(5) $$\phi_{M}\left(X_{i},Y_{j}\right)=\frac{M}{2}\left(\frac{1}{k}\sum_{\ell =1}^{k}\mu_{i\ell }+\frac{1}{m}\sum_{\ell =1}^{m}\mu_{\ell j}\right),$$

and

(6) $$\phi_{H}\left(X_{i},Y_{j}\right)=\frac{C}{2}\left(\frac{1}{k}\sum_{\ell =1}^{k}\eta_{i\ell }+\frac{1}{m}\sum_{\ell =1}^{m}\eta_{\ell j}\right)+\frac{C}{k\left(k+1\right)}\left(\gamma_{i}-\frac{\sum_{i=1}^{m}\gamma_{i}}{m}\right),$$

respectively. The formulations of $\phi_{M}\left(X_{i},Y_{j}\right)$ and $\phi_{H}\left(X_{i},Y_{j}\right)$ are obtained by decomposing the individual Shapley values of bacterial group $X_{i}$ and archaeal group $Y_{j}$ into pairwise bacteria–archaea interpretations. This decomposition does not change the individual Shapley values in Equations (3) and (4); it only provides a pairwise summary for ecological interpretation. The equal division across possible partners is used as a neutral baseline because independent pair‐specific rates, metabolite exchange measurements, isotope data, thermodynamic constraints, or gene‐expression data are not available. Under this baseline, the Shapley value of bacterial group $X_{i}$ is divided across its k possible archaeal partners, and the Shapley value of archaeal group $Y_{j}$ is divided across its m possible bacterial partners.

Under the non‐uniform decomposition, methane Shapley contributions are allocated across bacteria–archaea pairs according to the marginal interaction weights $\mu_{\cdot j}=\sum_{i=1}^{m}\mu_{\mathrm{ij}}$ and $\mu_{i\cdot }=\sum_{j=1}^{k}\mu_{\mathrm{ij}}$, while hydrocarbon contributions are allocated analogously using $\eta_{\cdot j}=\sum_{i=1}^{m}\eta_{\mathrm{ij}}$ and $\eta_{i\cdot }=\sum_{j=1}^{k}\eta_{\mathrm{ij}}$. Hence, for each pairwise coalition $\left(X_{i},Y_{j}\right)$, the methane and hydrocarbon Shapley contributions under the non‐uniform allocation are defined as follows:

(7) $$\phi_{M}\left(X_{i},Y_{j}\right)=\frac{M}{2}\left(\sum_{i=1}^{m}\mu_{\mathrm{ij}}\sum_{\ell =1}^{k}\mu_{i\ell }+\sum_{j=1}^{k}\mu_{\mathrm{ij}}\sum_{\ell =1}^{m}\mu_{\ell j}\right),$$

and

(8) $$\begin{array}{cc} \phi_{H}\left(X_{i},Y_{j}\right)= & \;\frac{C}{2}\left(\sum_{i=1}^{m}\eta_{\mathrm{ij}}\sum_{\ell =1}^{k}\eta_{i\ell }+\sum_{j=1}^{k}\eta_{\mathrm{ij}}\sum_{\ell =1}^{m}\eta_{\ell j}\right)\\ & \;+\frac{C}{k+1}\left(\gamma_{i}\sum_{i=1}^{m}\eta_{\mathrm{ij}}-\frac{1}{k}\sum_{j=1}^{k}\eta_{\mathrm{ij}}\sum_{i=1}^{m}\gamma_{i}\right), \end{array}$$

respectively. The weighted formulation therefore differs fundamentally from the uniform baseline. Under the uniform interpretation, each microbial group distributes its Shapley contribution equally among all potential partners. Under the weighted interpretation, this contribution is preferentially assigned toward partners having greater marginal importance in the inferred methane or hydrocarbon interaction structure. Consequently, pairs involving microbial groups with stronger inferred ecological contributions receive larger pairwise values, whereas associations involving groups with relatively weak inferred contributions receive smaller values. Since this study focuses on pairwise bacteria–archaea interpretations, higher‐order coalition decompositions are not considered further.

The coefficients of the Shapley values, namely $\gamma_{i}$, $\mu_{\mathrm{ij}}$, and $\eta_{\mathrm{ij}}$, play a critical role in determining microbial roles within methane‐hydrocarbon dynamics during the biodegradation process. In this study, we utilize 16S rRNA gene relative abundance for bacterial and archaeal groups to quantify their relative abundances at the initial stage of the process and to track changes in their abundance proportions at the final stage. These relative abundance dynamics are used to calibrate the coefficient values, ensuring that the payoff structure accurately reflects the underlying microbial composition and its temporal evolution. Specifically, let $b_{i}^{0}$ and $b_{i}^{1}$ denote the initial and final abundance percentages of bacterial group for $i\in \left\{1,2,\ldots ,m\right\}$, respectively. Similarly, let $a_{j}^{0}$ and $a_{j}^{1}$ denote the initial and final abundance percentages of archaeal group for $j\in \left\{1,2,\ldots ,k\right\}$, respectively. We now introduce a novel Shapley‐based interpretation to analyse methane–hydrocarbon dynamics across different regime changes. This framework enables us to quantify how microbial group contributions to methane production and hydrocarbon consumption evolve between initial and final stages of the biodegradation process. Accordingly, we define regime‐based Shapley reaction intensities for methane and hydrocarbon levels as follows:
(9)

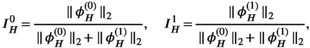

and
(10)

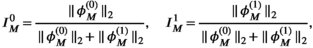

where $\phi_{M}^{\left(i\right)}$ and $\phi_{H}^{\left(i\right)}$ are $\left(m+k\right)$‐dimensional vectors that jointly collect the Shapley methane and hydrocarbon components of all bacterial and archaeal groups with $i\in \left\{0,1\right\}$ respectively, and $∥\cdot {∥}_{2}$ denotes the Euclidean norm. Representing the Shapley values of all microbial groups as regime‐specific vectors and comparing these vectors across incubation phases, quantifies shifts in collective functional intensity. With this way, we characterize how the distribution of microbial contributions to hydrocarbon consumption and methane production reorganizes between ecological regimes.

To characterize bacterial interactions during the initial stage of hydrocarbon consumption individually, we define γ as follows:

$$\gamma_{i}=\frac{b_{i}^{0}}{100},$$

for $i\in \left\{1,2,\ldots ,m\right\}$. Next, we define the following normalized enrichment terms to represent the relative change of bacterial and archaeal groups from the initial to the final phase:

$$\xi_{b_{i}}=\frac{\frac{b_{i}^{1}}{b_{i}^{0}}}{\sum_{\ell =1}^{m}\frac{b_{\ell }^{1}}{b_{\ell }^{0}}},\;\xi_{a_{j}}=\frac{\frac{a_{j}^{1}}{a_{j}^{0}}}{\sum_{\ell =1}^{k}\frac{a_{\ell }^{1}}{a_{\ell }^{0}}},$$

for $i\in \left\{1,2,\ldots ,m\right\}$ and $j\in \left\{1,2,\ldots ,k\right\}$, respectively. These quantities are abundance‐informed weights derived from the observed changes in 16S rRNA gene relative abundance between the two measured endpoints of each regime. They are therefore not interpreted as independently measured metabolic‐rate constants, but as ecological weights used to parameterize the cooperative game.

Hence, to represent the bacterial–archaeal methane‐related interaction coefficients $\mu_{\mathrm{ij}}$, we define them as the product of $\xi_{b_{i}}$, associated with the ith bacterial group, and $\xi_{a_{j}}$, associated with the jth archaeal group:
(11)

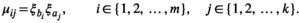




This formulation reflects the cooperative nature of methanogenic hydrocarbon consumption, in which bacterial groups contribute to hydrocarbon activation and fermentation, while archaeal groups participate in terminal methanogenic processes. Thus, $\mu_{\mathrm{ij}}$ provides an abundance‐informed approximation of the potential bacterial–archaeal coupling represented in the game.

Moreover, to construct the hydrocarbon‐related coefficients $\eta_{\mathrm{ij}}$ in the game value, we follow the classical syntrophic interpretation of anaerobic hydrocarbon biodegradation, where bacterial groups are treated as the main hydrocarbon‐transforming players and archaeal methanogens can indirectly support continued turnover through downstream consumption of fermentation products (Harindintwali et al. 2023; Siddique et al. 2012). Specifically, each coefficient $\eta_{\mathrm{ij}}$ is defined as a function of the bacterial contribution $\xi_{b_{i}}$, scaled by the combined contribution of the interacting bacterial and archaeal groups. More precisely, we define:

(12) $$\eta_{\mathrm{ij}}=\frac{{\tilde{\eta }}_{\mathrm{ij}}}{\sum_{i=1}^{m}\sum_{j=1}^{k}{\tilde{\eta }}_{\mathrm{ij}}},\;\text{where}\;{\tilde{\eta }}_{\mathrm{ij}}=\xi_{b_{i}}\left(\xi_{b_{i}}+\xi_{a_{j}}\right).$$

This construction gives primary weight to the bacterial term $\xi_{b_{i}}$, while still allowing the associated archaeal term $\xi_{a_{j}}$ to influence the bacteria–archaea interaction strength. The construction of $\mu_{\mathrm{ij}}$ and $\eta_{\mathrm{ij}}$ respects the required weight conditions in Equation (2).

To evaluate whether the resulting Shapley allocations depend strongly on proportional enrichment alone, we introduced an enrichment–prevalence sensitivity parameter $\lambda \in \left[0,1\right]$. For each bacterial and archaeal group, the sensitivity versions of $\xi_{b_{i}}$ and $\xi_{a_{j}}$ are defined as:
(13)

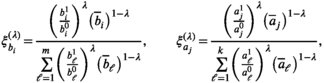

where

$${\bar{b}}_{i}=\frac{b_{i}^{0}+b_{i}^{1}}{2},\;{\bar{a}}_{j}=\frac{a_{j}^{0}+a_{j}^{1}}{2},$$

for $i\in \left\{1,2,\ldots ,m\right\}$ and $j\in \left\{1,2,\ldots ,k\right\}$. Here, λ=1 recovers the original normalized fold‐change formulation used in the main model, whereas λ=0 gives a prevalence‐oriented formulation based on the average relative abundance across the two observed endpoints of the regime. Intermediate values 0<λ<1 balance proportional enrichment with endpoint prevalence.

From an Environmental Microbiology perspective, this sensitivity analysis distinguishes microbial groups that become strongly enriched from low initial abundance from those that remain consistently represented within the community during the degradation interval. Thus, it tests whether the model allocations are driven primarily by proportional enrichment or whether the same dominant groups are retained when sustained community prevalence is also considered.

### 2.3 Stochastic Forecasting of Methane–Hydrocarbon Dynamics

This section presents a stochastic forecasting framework that integrates regime‐based Shapley reaction intensities within methane‐hydrocarbon dynamics in late‐stage anaerobic microbial tailings. The primary objective of this design is to capture both predicted hydrocarbon–methane trajectories and their associated uncertainty envelopes in order to establish a mechanistically interpretable bridge between microbial ecology and methane production behaviour.

Algorithm 1 shows the complete scheme of the forecasting, consisting of four primary steps. Initially, we define our input data as follows: we have data for observed methane ($M\left(t_{i}\right)$) and hydrocarbon ($H\left(t_{i}\right)$) concentrations for $i\in \left\{1,\cdots ,N\right\}$, along with $\left(m+k\right)$‐dimensional Shapley vectors for hydrocarbon and methane components, denoted $\phi_{H}^{\left(r\right)}$ and $\phi_{M}^{\left(r\right)}$, respectively, for the initial‐stage (r=1) and late‐stage (r=2) regimes. Additional inputs include the time of regime shift $T_{s}$ to determine the system shift from 1 to 2, noise parameters for both hydrocarbons and methane ($\sigma_{H0},\sigma_{H1},\sigma_{M0},\sigma_{M1}$), a late‐stage starting time $T_{N}$, the number of Monte Carlo simulations $N_{\mathrm{MC}}$, the simulation time step Δt, and the number of forecast steps K.

First step involves determining the Shapley reaction intensities $I_{H}^{\left(r\right)}$ and $I_{M}^{\left(r\right)}$, which are later utilized in the deterministic fitting and stochastic forecasting components to enable the model to integrate the regime‐dependent characteristics of microbial interactions and biochemical dynamics. Two ecological regimes (r∈1,2) are defined based on temporal shifts observed in 16S rRNA gene community profiles. The early‐stage regime (r=1) extends from 0 to 400 days, representing the initial phase of anaerobic microbial establishment. In contrast, the late‐stage regime (r=2), which spans 400–900 days, indicates a restructured and functionally stabilized community linked to sustained methanogenic activity. In both stages, $\left(m+k\right)$‐dimensional Shapley vectors $\left(X_{1},X_{2},\cdots ,X_{m},Y_{1},Y_{2},\cdots ,Y_{k}\right)$ are computed for hydrocarbon and methane components utilizing Equations (3) and (4). Then, Equations (9) and (10) are used to derive Shapley reaction intensities for both regimes.

Second step uses the deterministic drift components of hydrocarbon and methane changes under Shapley regimes using Lasso regression. Here, we first arrange Shapley regimes according to given switching time $T_{s}$ Here, we construct feature vectors

$$X_{i}=\left[H\left(t_{i}\right),M\left(t_{i}\right),I_{H}^{\left(r\right)},I_{M}^{\left(r\right)}\right]$$

and fit Lasso‐regularized linear models: $f_{j}\left(X_{i}\right)=\beta_{j,0}+\beta_{j}^{⊤}X_{i},$ where coefficients are obtained by solving:

$${\hat{\beta }}_{j}=\arg\min_{\beta }\left(\sum_{i}{\left(\Delta j_{i}-\beta_{j,0}-\beta^{⊤}X_{i}\right)}^{2}+\lambda_{j}∥\beta {∥}_{1}\right)$$

where $j\in \left\{H,M\right\}$. Lasso is employed because hydrocarbon consumption and methane production can be approximated as first‐order, near‐linear responses to current substrate levels within each regime. By incorporating interaction intensities into the feature vectors, Lasso isolates the dominant linear drift terms that couple hydrocarbon consumption to methane generation while suppressing secondary effects.

Third step provides late‐stage (r=2) stochastic forecast by using time‐series approach and Monte Carlo simulations. For each realization i out of $N_{\mathrm{MC}}$ simulations, the system is initialized at the last‐stage starting time $T_{N}$ for both hydrocarbon and methane components and iteratively propagates the system forward for K forecast steps, using time step Δt. The stochastic components of hydrocarbon and methane changes are computed by combining the Lasso‐determined drift with additive Gaussian noise, whose variance is scaled according to the regime‐specific intensities: $\sigma_{H}=\sigma_{H0}+\sigma_{H1}I_{H}^{\left(r\right)}$ and $\sigma_{M}=\sigma_{M0}+\sigma_{M1}I_{M}^{\left(r\right)}$. Additionally, we conduct random draws $\varepsilon_{H}$ and $\varepsilon_{M}$ from normal distributions with specified variances to incorporate stochasticity presented in microbial interactions.

The next hydrocarbon data ($H_{i}^{\left(k+1\right)}$) is updated by deducting the current hydrocarbon data ($H_{i}^{\left(k\right)}$) from the absolute value of the combined deterministic term (${\mathrm{dH}}_{\det}$) associated with the late‐stage Shapley reaction regime ($I_{H}^{\left(2\right)}$) and the stochastic term $\varepsilon_{H}$. This process employs the max operator to maintain non‐negativity, stopping the iteration when the difference yields a negative value. With this setting, we establish hydrocarbon depletion as influenced by the late‐stage regime, which affects both the depletion magnitude and the randomness arising from late‐stage microbial interactions. On the other hand, the subsequent methane data ($M_{i}^{\left(k+1\right)}$) are updated by incorporating the term, $|{\mathrm{dM}}_{\det}+\varepsilon_{M}|S_{k}$, where $S_{k}=H_{i}^{\left(k+1\right)}/H_{0}$, into the existing methane data ($M_{i}^{\left(k\right)}$). This adjustment illustrates the relationship between methane production and the current hydrocarbon availability, as well as late‐stage microbial uncertainty involved in methane production. Finally, the fourth step computes the mean hydrocarbon and methane trajectories, $\bar{H}\left(t_{k}\right)$ and $\bar{M}\left(t_{k}\right)$, after completing all $N_{\mathrm{MC}}$ realizations. Then, we calculate the 10th and 90th percentiles of the Monte Carlo ensembles, yielding confidence bands $H_{\mathrm{low}/\text{high}}\left(t_{k}\right)$ and $M_{\mathrm{low}/\text{high}}\left(t_{k}\right)$ that capture the likely variability in hydrocarbon and methane levels.

ALGORITHM 1 **Shapley‐Based Methane–Hydrocarbon Forecasting.**

1: **Input:** Observed data $\left\{H\left(t_{i}\right),M\left(t_{i}\right)\right\}$, microbial Shapley vectors $\phi_{H}^{\left(r\right)},\phi_{M}^{\left(r\right)}$ for regimes $r\in \left\{1,2\right\}$, regime switching time $T_{s}$, late-stage starting time $T_{N}$, noise parameters $\sigma_{H0},\sigma_{H1},\sigma_{M0},\sigma_{M1}$, Monte Carlo size $N_{\mathrm{MC}}$, step size Δt, forecast steps K.2: **Step 1: Compute Shapley Reaction Intensities**3: Determine: $I_{H}^{\left(r\right)}$ and $I_{M}^{\left(r\right)}$4: **Step 2: Fit Regime‐based Deterministic Drift**5: **for** each observed interval $\left(t_{i},t_{i+1}\right)$ **do**6:  Determine regime:                   $r=\left(\begin{array}{ll} 1 & \text{if}\;t_{i}<T_{s},\\ 2 & \text{if}\;t_{i}\geq T_{s} \end{array}\right.$7:  Construct feature vector: $X_{i}=\left[H\left(t_{i}\right),M\left(t_{i}\right),I_{H}^{\left(r\right)},I_{M}^{\left(r\right)}\right]$8:  Target responses: $\Delta H_{i}=H\left(t_{i+1}\right)-H\left(t_{i}\right),\;\Delta M_{i}=M\left(t_{i+1}\right)-M\left(t_{i}\right)$9: **end for**10: Fit Lasso models: $\Delta H\approx f_{H}\left(X_{i}\right)$;    $\Delta M\approx f_{M}\left(X_{i}\right)$11: **Step 3: Late‐stage Stochastic Forecast**12: **for** i=1 to $N_{\mathrm{MC}}$ **do**13:  Initialize:          $H_{i}^{\left(0\right)}=H\left(t_{N}\right),\;M_{i}^{\left(0\right)}=M\left(t_{N}\right),\;t_{0}=t_{N}$14:  **for** k=0 to K−1 **do**15:    Fix late-stage regime: r=216:    Compute late-stage noise amplitudes: $\sigma_{H}=\sigma_{H0}+\sigma_{H1}I_{H}^{\left(2\right)},\;\sigma_{M}=\sigma_{M0}+\sigma_{M1}I_{M}^{\left(2\right)}$17:    Sample late-stage stochastic perturbations: $\varepsilon_{H}∼N\left(0,\sigma_{H}^{2}\right),\;\varepsilon_{M}∼N\left(0,\sigma_{M}^{2}\right)$18:    Deterministic drifts: ${\mathrm{dH}}_{\det}=f_{H}\left(H_{i}^{\left(k\right)},M_{i}^{\left(k\right)},I_{H}^{\left(2\right)},I_{M}^{\left(2\right)}\right);{\mathrm{dM}}_{\det}=f_{M}\left(H_{i}^{\left(k\right)},M_{i}^{\left(k\right)},I_{H}^{\left(2\right)},I_{M}^{\left(2\right)}\right)$19:    Late-stage hydrocarbon update: $H_{i}^{\left(k+1\right)}=\max\left(H_{i}^{\left(k\right)}-\left|I_{H}^{\left(2\right)}{\mathrm{dH}}_{\det}+\varepsilon_{H}\right|,0\right)$20:    Late-stage methane update: $M_{i}^{\left(k+1\right)}=M_{i}^{\left(k\right)}+|{\mathrm{dM}}_{\det}+\varepsilon_{M}|\;S_{k},\;\text{where}\;S_{k}=\frac{H_{i}^{\left(k+1\right)}}{H_{0}}$21:    $t_{k+1}=t_{k}+\Delta t$22:  **end for**23:  **end for**24: **Step 4: Monte Carlo Aggregation**        $\bar{H}\left(t_{k}\right)=\frac{1}{N_{\mathrm{MC}}}\sum_{i=1}^{N_{\mathrm{MC}}}H_{i}\left(t_{k}\right),\;\bar{M}\left(t_{k}\right)=\frac{1}{N_{\mathrm{MC}}}\sum_{i=1}^{N_{\mathrm{MC}}}M_{i}\left(t_{k}\right)$        $H_{\mathrm{low}/\text{high}}\left(t_{k}\right)=P_{10/90}\;\left(\left\{H_{i}\left(t_{k}\right)\right\}\right),\;M_{\mathrm{low}/\text{high}}\left(t_{k}\right)=P_{10/90}\;\left(\left\{M_{i}\left(t_{k}\right)\right\}\right)$25: **Output:** $\bar{H}\left(t\right),H_{\mathrm{low}/\text{high}}\left(t\right),\bar{M}\left(t\right),M_{\mathrm{low}/\text{high}}\left(t\right)$

To test whether the Shapley‐based forecasting framework provides information beyond simpler alternatives, we performed an ablation analysis using the same calibration period, 0–1300 days, and the same independent Day–1600 validation point used in the main forecast. The proposed model is referred to here as the full Shapley‐based forecast model. It uses the feature vector

$$X_{i}=\left[H\left(t_{i}\right),M\left(t_{i}\right),I_{H}^{\left(r\right)},I_{M}^{\left(r\right)}\right],$$

where $H\left(t_{i}\right)$ and $M\left(t_{i}\right)$ are the observed hydrocarbon concentration and methane production, and $I_{H}^{\left(r\right)}$ and $I_{M}^{\left(r\right)}$ are regime‐specific Shapley reaction intensities for hydrocarbon consumption and methane production. In the ablation analysis, two regimes are used: the initial regime, r=1, for $t_{i}<600$ days, and the late regime, r=2, for $t_{i}\geq 600$ days. The Day–1600 forecast is therefore generated under the late‐stage regime, r=2.

We compared the full Shapley‐based forecast model with the following benchmarks:

- **Shapley linear‐regression benchmark:** uses the same Shapley‐based feature vector as the full model, but replaces Lasso regression with ordinary linear regression.
- **Abundance‐only Lasso benchmark:** keeps the Lasso forecasting structure, but replaces Shapley reaction intensities with endpoint‐abundance‐derived intensities. This tests whether abundance information alone can explain the Day–1600 forecast.
- **State‐only Lasso ablation:** uses only the process variables $\left[H\left(t_{i}\right),M\left(t_{i}\right)\right]$ and removes all microbial and regime‐intensity terms.
- **Regime‐only Lasso ablation:** uses $\left[H\left(t_{i}\right),M\left(t_{i}\right),I\left(r=2\right)\right]$, where $I\left(r=2\right)$ indicates the late regime. This tests whether simply distinguishing the initial and late regimes is sufficient without Shapley or abundance‐based microbial intensities.
- **Time‐only linear regression benchmark:** predicts hydrocarbon and methane directly as linear functions of time, without microbial information or stochastic simulation
- **Logistic/saturation benchmark:** fits continuous saturation‐type curves to hydrocarbon depletion and methane accumulation.
- **Null persistence benchmark:** assumes no change after Day–1300, so the Day–1600 prediction equals the Day–1300 observed value.

Model performance is evaluated using the Day–1600 predicted hydrocarbon and methane values and their relative errors.

### 2.4 Overall Workflow

The overall workflow integrates the microbial community, hydrocarbon, and methane data described in Section 2.1 with the Shapley cooperative game formulation introduced in Section 2.2 and the forecasting framework described in Section 2.3. The goal is to quantify regime‐dependent bacterial–archaeal contributions to coupled hydrocarbon consumption and methane production in anaerobic oil sands tailings. For each site, the analysis uses 16S rRNA gene relative abundance, hydrocarbon concentrations, and methane concentrations as joint inputs. The microbial Shapley analysis is based on two 16S‐defined incubation regimes: an initial microbial regime from 0 to 400 days and a late microbial regime from 400 to 900 days. These two regimes are used to construct the microbial interaction coefficients and Shapley contributions because they correspond to the available microbial community sampling intervals.

As shown in Figure 1, bacterial and archaeal groups are treated as players in a cooperative game within each regime. Individual and pairwise Shapley values are calculated to quantify contributions to methane production and hydrocarbon consumption. Individual effects are evaluated using Equations (3) and (4), whereas pairwise coalition effects are evaluated using Equations (5) and (6). This step identifies dominant taxa and cross‐domain partnerships based on relative abundance data. The resulting Shapley values are then mapped to methane and hydrocarbon reaction intensities through Equations (9) and (10), providing the link between microbial attribution and dynamic prediction.

**FIGURE 1:**
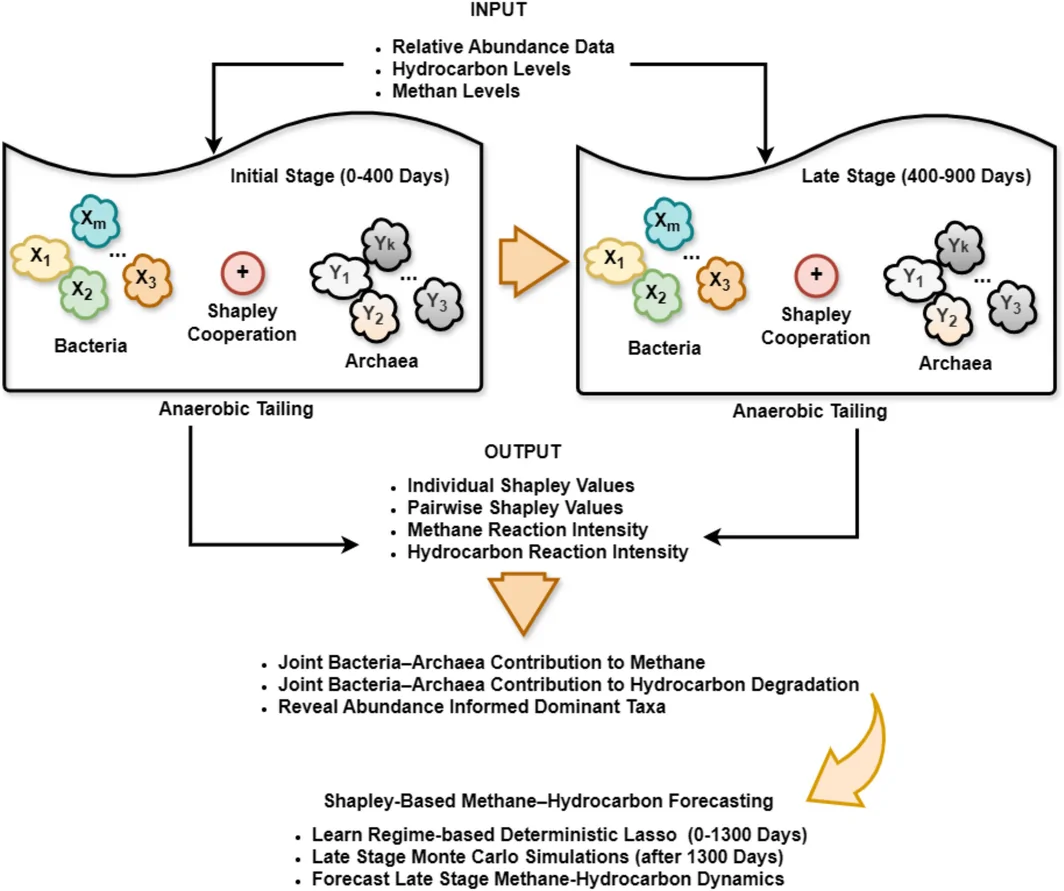
Integrated analytical workflow for Shapley‐based attribution and forecasting in anaerobic oil sands tailings. Relative abundance data, hydrocarbon concentrations, and methane concentrations are evaluated across initial and late‐stage regimes. Individual and pairwise Shapley values are used to quantify bacterial and archaeal contributions to hydrocarbon consumption and methane production. These outputs are converted into reaction intensities and incorporated into deterministic Lasso, Monte Carlo simulation, and time‐series forecasting; the forecasting switch at 600 days assigns the hydrocarbon–methane observations to initial or late forecasting states, while the microbial Shapley regimes remain defined by the 0–400 and 400–900 day 16S intervals.

Microbial regimes are distinct from the forecasting switch time used in the dynamic prediction step. The 0–400 and 400–900 day intervals define the microbial interaction structure, whereas the forecasting switch time determines how the observed hydrocarbon–methane time series is assigned to initial or late forecasting states. Thus, the forecasting switch does not introduce a new microbial 16S regime; it only controls how the fitted Lasso model uses the initial and late Shapley‐derived reaction intensities.

For forecasting, both sites are evaluated using observed methane and hydrocarbon data at 0, 400, 600, 900, and 1300 days, giving N=5 observation times. In the case‐study implementation, the forecasting switch time is set to $T_{s}=600$ days. This means that observations before 600 days are assigned to the initial forecasting state, while observations from 600 days onward are assigned to the late forecasting state. This choice uses the observed 600‐day process measurement as the transition point between early and later hydrocarbon–methane dynamics and is consistent with the staged biodegradation behaviour reported for these incubations (Shahimin and Siddique 2017). The late forecasting state uses the Shapley‐derived reaction intensities obtained from the 400–900 day microbial regime. The value $T_{N}=1300$ days denotes the final calibration time and the starting point for forward prediction; it is not a separate microbial regime. After 1300 days, the forecast assumes persistence of the late‐stage Shapley structure.

The forecasting setup uses a temporal step size of Δt=100 days, a forecast horizon of K=15 steps, and $N_{\mathrm{MC}}=500$ Monte Carlo realizations. Hydrocarbon noise parameters are set to $\sigma_{H0}=80$ and $\sigma_{H1}=120$, whereas methane noise parameters are set to $\sigma_{M0}=0.05$ and $\sigma_{M1}=0.5$. Deterministic Lasso is first fitted to the observed methane and hydrocarbon data over 0–1300 days to characterize regime‐dependent dynamics. These fitted dynamics are then propagated through Monte Carlo simulation and time‐series forecasting to predict methane generation and hydrocarbon consumption after 1300 days at both sites.

## 3 Numerical Results

This section summarizes the numerical results obtained from applying the Shapley framework to the ALBIAN and CNRL MFT systems described in Section 2.1. Table 3 compares the percentage contributions to methane production, as estimated by the Shapley approach, with the corresponding 16S rRNA relative abundances for the dominant microbial groups in naphtha‐amended microcosms. Results for ALBIAN are shown in Panel A and for CNRL in Panel B. For each site and incubation regime, Shapley values are first calculated individually for every microbial group. These values are then normalized within each phase so that total contributions to methane sum to 100% for the early (0–400 days) and late (400–900 days) stages. Microbial relative abundance values are taken at 400 days to represent the initial stage and at 900 days to represent the late stage, as reported in Table 1.

**TABLE 3:** Comparison of percentage Shapley contributions to methane production and 16S rRNA gene relative abundance (%) for microbial groups in naphtha‐amended MFT microcosms from ALBIAN (Panel A) and CNRL (Panel B).

Microbial group	0–400 Days		400–900 Days	
	Shapley (%)	Abundance (%)	Shapley (%)	Abundance (%)
Panel A: ALBIAN				
Bacteria				
Betaproteobacteria	0.00	3.2	2.70	0.5
Anaerolineae	2.35	27.0	5.50	8.6
Clostridia	47.19	38.8	34.91	78.5
Others	0.46	31.0	6.90	12.4
Archaea				
Methanosaetaceae	3.50	60.8	5.70	28.1
Methanosarcinaceae	0.54	2.3	7.51	1.4
Methanoregula	45.08	32.3	25.27	66.2
Others	0.89	4.6	11.53	4.3
Panel B: CNRL				
Bacteria				
Actinobacteria	2.83	7.5	0.01	1.2
Anaerolineae	9.67	31.1	0.00	2.0
Clostridia	0.39	0.1	49.90	65.2
Deltaproteobacteria	31.54	24.6	0.06	19.4
Others	5.60	36.7	0.03	12.2
Archaea				
Methanosaetaceae	31.07	80.6	0.88	8.1
Methanoregula	15.38	17.3	46.60	91.3
Others	3.55	2.1	2.52	0.6

*Note:* Shapley values are normalized across all microbial groups within each incubation regime such that contributions sum to 100% for methane in each phase (0–400 and 400–900 days). Abundance values are presented at 400 days for the initial‐stage and 900 days for the late‐stage.

During the early phase at ALBIAN, methane production is primarily attributed to Clostridia and Methanoregula. Their Shapley‐inferred contributions are larger than would be suggested by abundance ranking alone, consistent with a syntrophic association between fermentative bacteria and hydrogenotrophic methanogens. In contrast, Anaerolineae is relatively abundant but receives only a small inferred methane contribution. A similar pattern occurs among archaea, where Methanosaetaceae remains abundant but is assigned a comparatively low contribution to methane production. In the later phase, relative abundance and Shapley attribution show greater agreement, with Clostridia and Methanoregula becoming dominant and receiving substantial inferred methane contributions, consistent with a more established hydrogenotrophic methanogenic network. However, the attribution does not fully mirror abundance ranking within the archaeal community: Methanosarcinaceae remains relatively rare but receives a larger inferred methane contribution than the more abundant Methanosaetaceae.

Unlike ALBIAN, the CNRL system shows a more acetoclastic pattern during the initial phase, with Methanosaetaceae receiving the largest inferred methane contribution. Methanoregula, although less abundant, also receives a substantial attribution relative to its community representation. A similar contrast occurs among bacteria: Deltaproteobacteria shows a comparatively large inferred contribution, whereas the more abundant Anaerolineae is assigned a smaller share of methane production. By the later stage, the CNRL community shifts toward a more syntrophically structured system, with methane production attributed predominantly to Clostridia and Methanoregula, both of which are highly abundant. In contrast, Methanosaetaceae retains a residual presence but receives only a minor inferred methane contribution. Likewise, Deltaproteobacteria, Actinobacteria, and Anaerolineae remain present in the bacterial community but are assigned comparatively small contributions to methane formation. Overall, the later‐stage pattern indicates a stronger concentration of model‐implied methane attribution within the Clostridia–Methanoregula coalition.

Figure 2 illustrates the enrichment–prevalence sensitivity analysis, supporting the methane Shapley patterns reported in Table 3 across alternative abundance‐weighted scenarios. The dashed λ=1 column reproduces the original normalized fold‐change results, while lower λ values test whether the same allocations persist when endpoint‐average relative abundance receives greater weight. In ALBIAN, Clostridia and Methanoregula remain the major methane‐associated groups under the original formulation and continue to receive substantial contributions across the tested λ range. In CNRL, the main patterns are also retained: Methanosaetaceae remains the dominant archaeal contributor during 0–400 days, while Deltaproteobacteria represents the strongest bacterial contributor under the original early‐stage formulation. During 400–900 days, Clostridia and Methanoregula consistently dominate methane attribution, including under alternative mean‐weighted scenarios.

**FIGURE 2:**
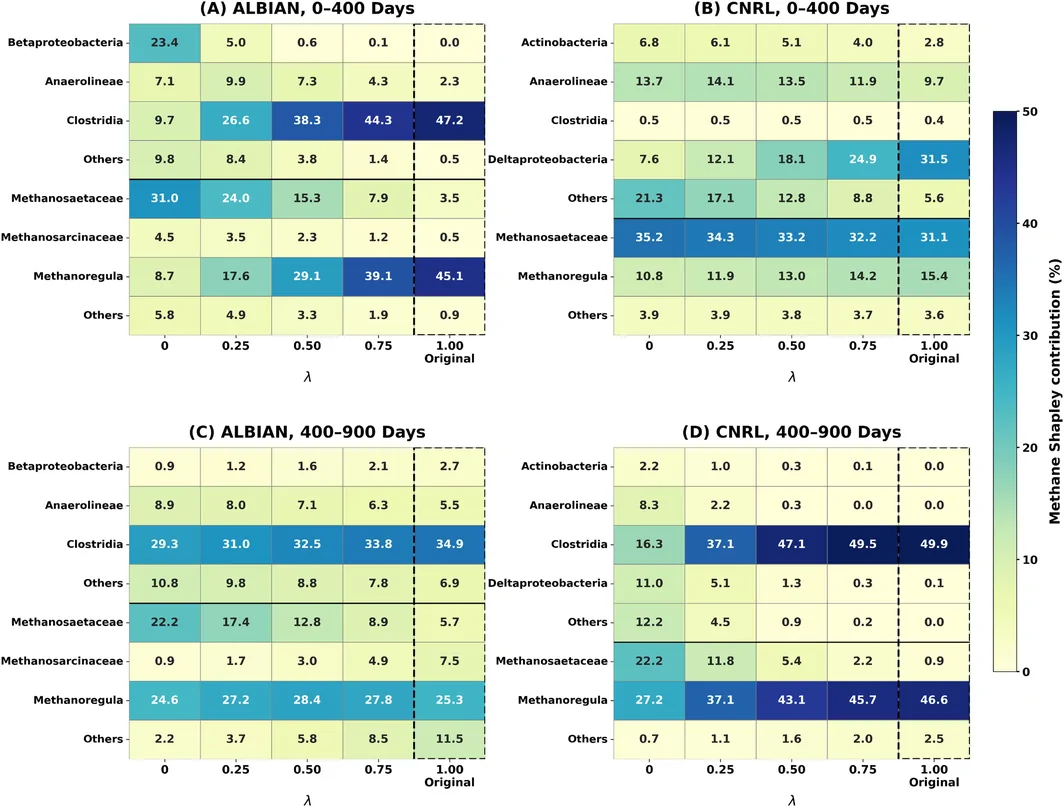
Sensitivity of methane Shapley contributions to the enrichment–prevalence parameter λ in ALBIAN and CNRL cases. Cell values show percentage methane Shapley contributions, normalized to sum to 100% within each site, period, and λ value. The dashed column indicates the original formulation (λ=1). Lower λ values emphasize endpoint‐average relative abundance, whereas higher values emphasize proportional enrichment between the two observed 16S rRNA gene sampling points.

Figure 3 reports Shapley values for pairwise coalitions between bacterial and archaeal groups in ALBIAN MFTs across initial‐stage and late‐stage time regimes based on the uniform allocation strategy given in Equations (5) and (6). Panels A and C show that the Clostridia–Methanoregula coalition is the dominant contributor to methane production; correspondingly, Clostridia and Methanoregula emerge as the most influential bacterial and archaeal contributors, respectively. This pattern persists into the late‐stage, although methane contributions decline substantially, with the Clostridia–Methanoregula coalition remaining the most impactful interaction. Consistent with these methane results, Panels B and D indicate that the Clostridia–Methanoregula coalition also provides the largest contribution to hydrocarbon consumption in both time regimes.

**FIGURE 3:**
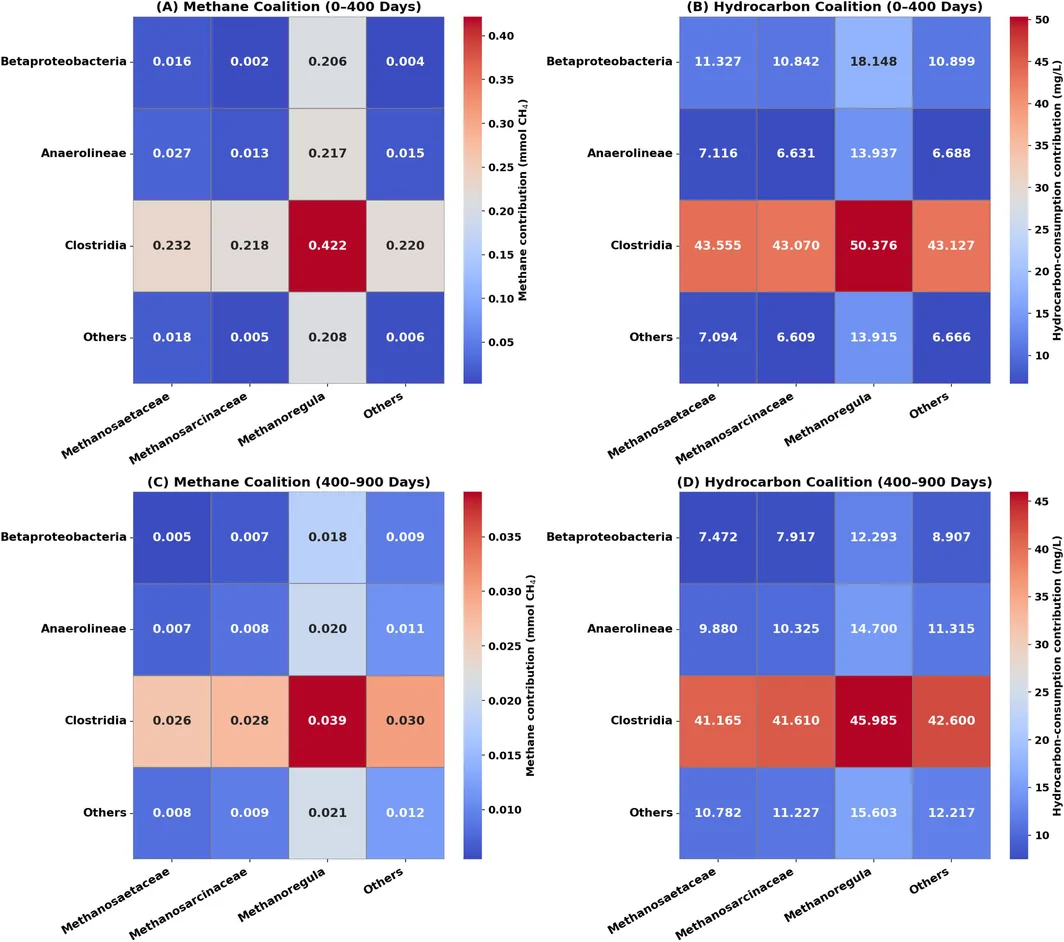
The heat maps of microbial Shapley coalition values for ALBIAN MFTs under the uniform allocation strategy. Colour gradients represent the magnitude of pairwise Shapley contribution. (A, C) Show methane contribution, $\phi_{M}\left(X_{i},Y_{j}\right)$, in mmol CH_4_, given by Equation (5) whereas (B, D) show hydrocarbon‐consumption contribution, $\phi_{H}\left(X_{i},Y_{j}\right)$, in mg/L, given by Equation (6). Each panel uses an independent colour scale because methane and hydrocarbon contributions have different units and regime‐specific numerical ranges; annotated cell values stand for direct quantitative comparison across panels.

On the other hand, Figure 4 shows a completely different scenario in the CNRL case under the uniform allocation strategy, where the most productive methane‐generating coalition is Deltaproteobacteria–Methanosaetaceae in the initial stage (see Figure 4A,B). However, this trend shifts toward a Clostridia‐centered network, with other archaea groups, mostly with the Methanoregula coalition in the late stage (see Figure 4C,D). By comparing two time regimes, we see that the behaviour of Clostridia in the ALBIAN case differs significantly from that in the CNRL case. The Clostridia–Methanoregula coalition exhibits minimal methane–hydrocarbon performance during the initial phase of CNRL but shows the most increased methane production activity in the later phase of CNRL, while this coalition is the most active in methane–hydrocarbon performance during the early phase of ALBIAN.

**FIGURE 4:**
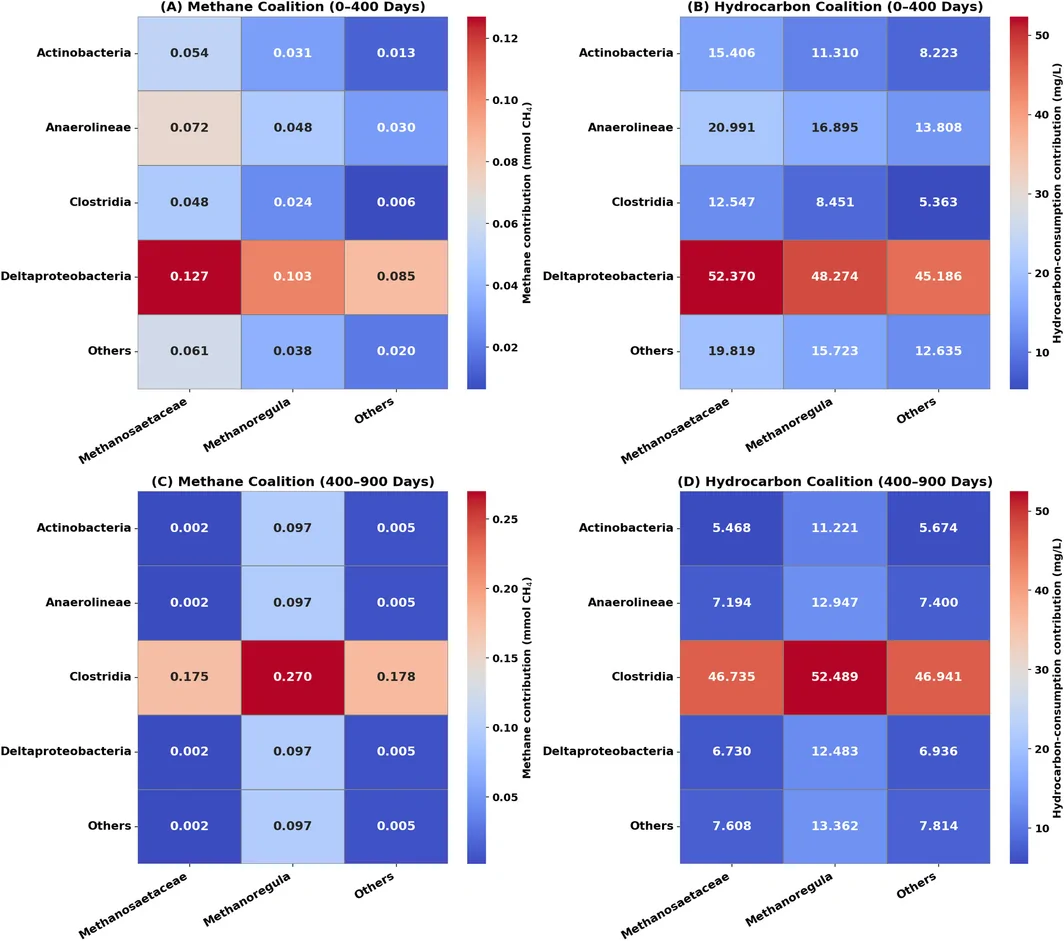
The heat maps of microbial Shapley coalition values in CNRL MFTs under the uniform allocation strategy. Colour gradients represent the magnitude of pairwise Shapley contribution. (A, C) Show methane contribution, $\phi_{M}\left(X_{i},Y_{j}\right)$, in mmol CH_4_, given by Equation (5) whereas (B, D) show hydrocarbon‐consumption contribution, $\phi_{H}\left(X_{i},Y_{j}\right)$, in mg/L, given by Equation (6). Each panel uses an independent colour scale because methane and hydrocarbon contributions have different units and regime‐specific numerical ranges; annotated cell values stand for direct quantitative comparison across panels.

Figures 5A–D and 6A–D illustrate the pairwise Shapley coalition contributions under the non‐uniform allocation strategy. The resulting patterns are qualitatively consistent with those obtained under the uniform allocation. For Albian, Clostridia–Methanoregula remains the dominant coalition for both methane production and hydrocarbon consumption across the two incubation periods. For CNRL, the dominant coalition shifts from Deltaproteobacteria–Methanosaetaceae during 0–400 days to Clostridia–Methanoregula during 400–900 days. This consistency indicates that the main ecological patterns are not driven by a particular pairwise allocation rule.

**FIGURE 5:**
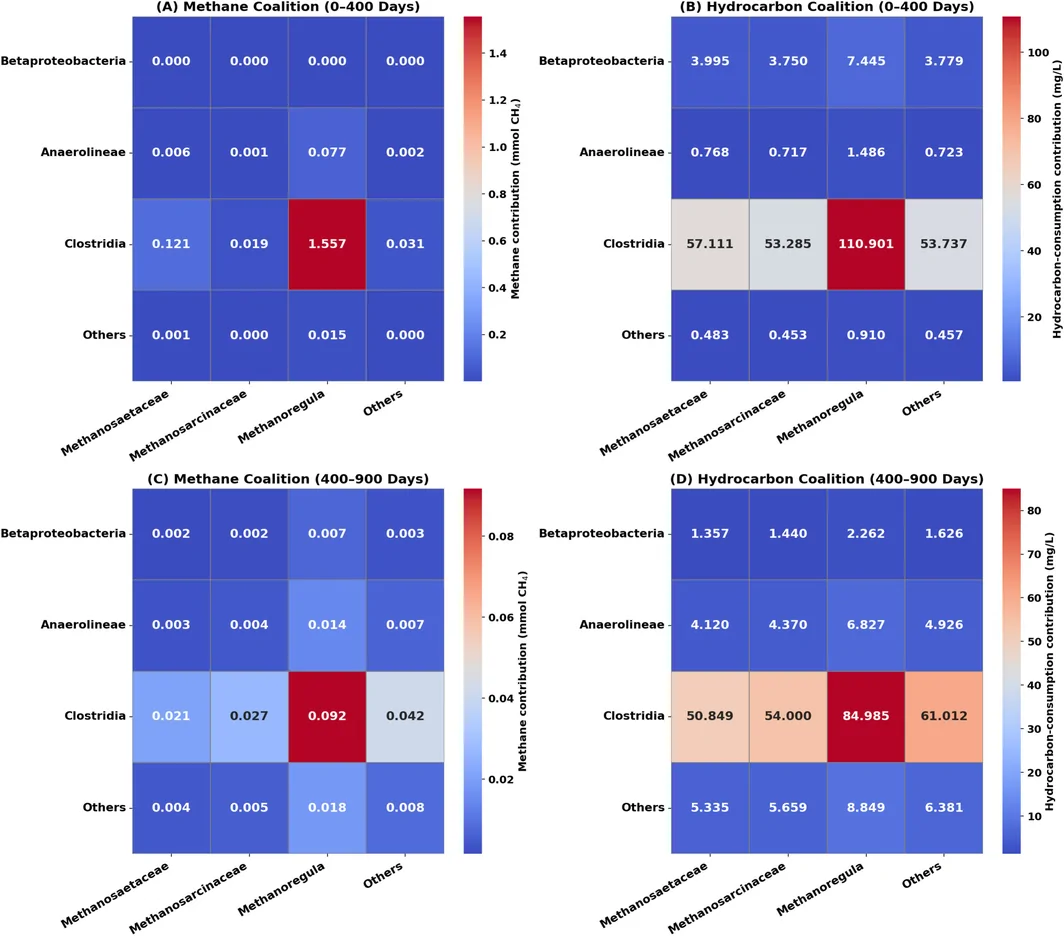
The heat maps of microbial Shapley coalition values for ALBIAN MFTs under the non‐uniform allocation strategy. Colour gradients represent the magnitude of pairwise Shapley contribution. (A, C) Show methane contribution, $\phi_{M}\left(X_{i},Y_{j}\right)$, in mmol CH_4_, given by Equation (7) whereas (B, D) show hydrocarbon‐consumption contribution, $\phi_{H}\left(X_{i},Y_{j}\right)$, in mg/L, given by Equation (8).

**FIGURE 6:**
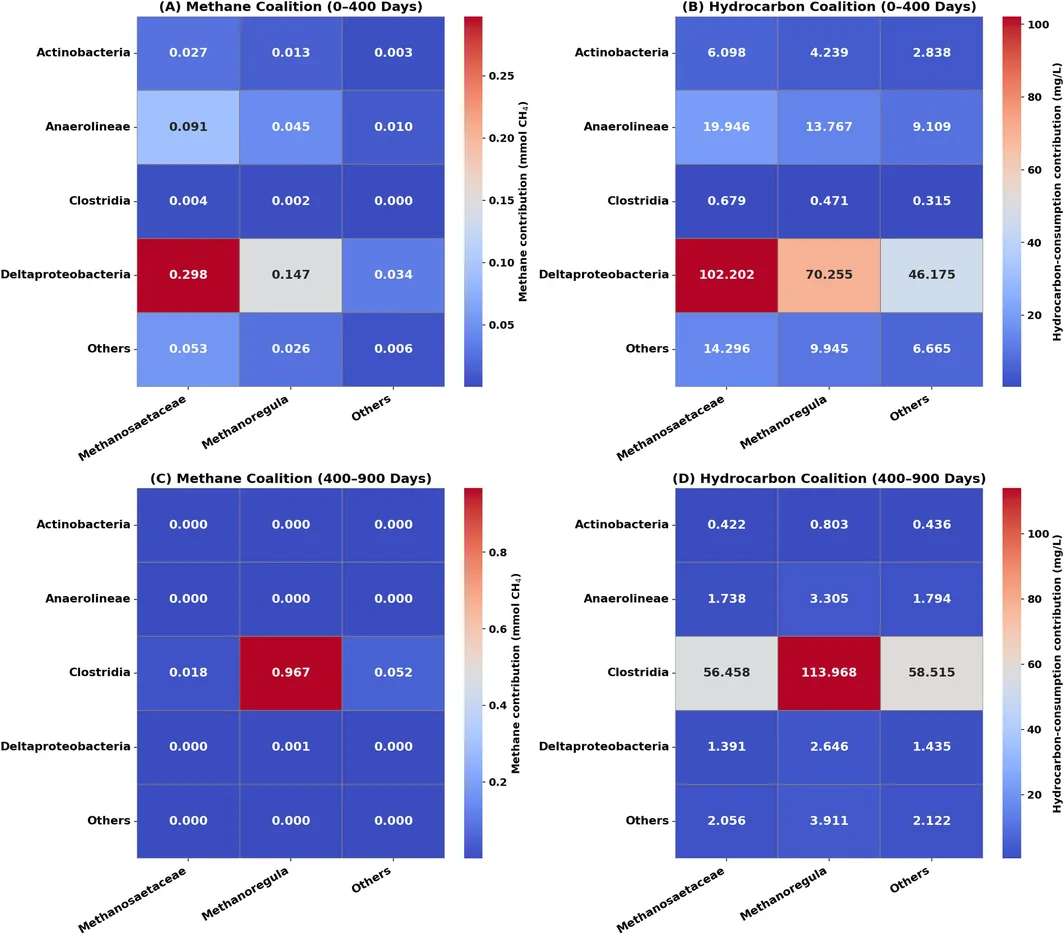
The heat maps of microbial Shapley coalition values in CNRL MFTs under the non‐uniform allocation strategy. Colour gradients represent the magnitude of pairwise Shapley contribution. (A, C) Show methane contribution, $\phi_{M}\left(X_{i},Y_{j}\right)$, in mmol CH_4_, given by Equation (7) whereas (B, D) show hydrocarbon‐consumption contribution, $\phi_{H}\left(X_{i},Y_{j}\right)$, in mg/L, given by Equation (8).

Table 4 presents the model calibration results from 0 to 1300 days, including the *R* ^2^ score and root mean square error (RMSE) for each site, as well as the model predictions at day 1600, which feature absolute and relative errors along with coverage information. The results indicate that Algorithm 1 exhibits high calibration performance and achieves highly accurate predictions of methane emissions in both scenarios. The validation performance for methane output, with a relative error near 3%, and hydrocarbon output, with a relative error close to zero, at 1600 days is notably accurate, and both lie within the percentile interval for the CNRL case. On the other hand, the ALBIAN case demonstrates prediction accuracy for hydrocarbon output, as it falls outside the percentile interval, with a relative error of 38%, while it accurately predicts methane output within the percentile interval, with an error of 4%. Overall, methane predictions at 1600 days from Algorithm 1 demonstrate significantly improved accuracy compared to the theoretical methane production estimates performed in Shahimin and Siddique (2017).

**TABLE 4:** Model calibration (0–1300 days) and independent Day–1600 out‐of‐sample validation results for hydrocarbon concentration (mg/L) and methane production (mmol/100 mL) in CNRL and ALBIAN microcosms.

Metric	CNRL		ALBIAN	
	Hydrocarbon	Methane	Hydrocarbon	Methane
Deterministic calibration (0–1300 days)				
*R* ^2^	1.000	0.996	1.000	0.998
RMSE	0.66	0.048	0.34	0.040
Day–1600 out‐of‐sample validation				
True value	1292.00	3.00	870.00	2.38
Predicted (Mean)	1293.08	3.09	540.20	2.49
Absolute error	1.08	0.088	329.80	0.105
Relative error (%)	0.08	2.95	37.91	4.42
Coverage (P10–P90)	Yes	Yes	No	Yes

*Note:* Deterministic fit metrics (*R* ^2^, RMSE) are computed over the calibration period. Forecast accuracy at Day–1600 is evaluated using the ensemble mean prediction. Relative error, calculated as the absolute difference between the predicted and true values divided by the true value, expressed as a percentage. Coverage indicates whether the observed value falls within the 10th–90th percentile predictive interval of the Monte Carlo ensemble.

Figure 7 presents the simulation outputs of Algorithm 1 for CNRL (Figure 7A,B) and ALBIAN (Figure 7C,D). Results show clear differences in both substrate degradation dynamics and methane generation patterns. In the late phase, CNRL exhibits a slower decline in hydrocarbon concentration compared to ALBIAN (Figure 7A,C). This suggests that the microbial consortia associated with CNRL exhibit sustained metabolic functionality compared to those associated with ALBIAN. Despite this slower late‐stage degradation, CNRL shows a pronounced increase in methane production (Figure 7D); notably, methane output nearly doubles relative to the 0–1300 day interval. In contrast, ALBIAN displays the late‐phase methane increases only modestly compared with its initial 0–1300 day stage (Figure 7B). This response may indicate earlier substrate exhaustion, reduced syntrophic efficiency, or a decline in methanogenic population density over time.

**FIGURE 7:**
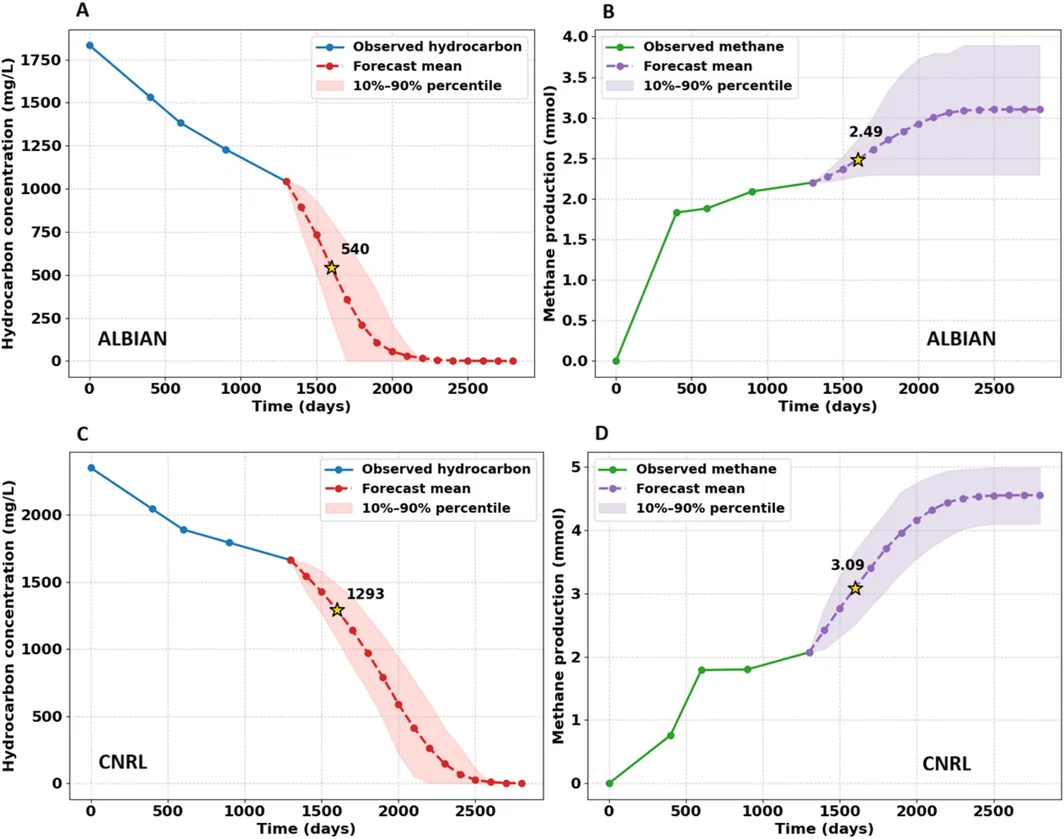
Time‐series calibration and forecasting of hydrocarbon consumption and methane production in naphtha‐amended MFT microcosms from ALBIAN (A, B) and CNRL (C, D). (A, C) Show observed hydrocarbon concentrations (solid blue) and model forecasts (red dashed) with 10%–90% predictive intervals (shaded). (B, D) Present observed methane production (solid green) and corresponding forecasts (purple dashed) with 10%–90% uncertainty bounds. A yellow star in each plot represents the estimated value at 1600 days.

Table 5 shows that our forecast model provides competitive or improved Day–1600 prediction compared with reduced and non‐microbial benchmarks. In CNRL, our model gives the best hydrocarbon prediction and also gives low methane error, outperforming the state‐only, regime‐only, time‐only, logistic/saturation, and persistence benchmarks. The abundance‐only Lasso benchmark gives a slightly lower methane error in CNRL, but it does not retain the Shapley‐based interaction structure used to attribute methane production and hydrocarbon consumption. In ALBIAN, our model improves hydrocarbon prediction compared with the abundance‐only, state‐only, and regime‐only microbial ablations, and gives low methane error. Although the time‐only and logistic/saturation regressions give lower ALBIAN hydrocarbon error, they perform worse for methane and do not include microbial interaction information. Hence, the ablation results indicate that the Shapley‐derived reaction intensities add useful forecasting information beyond state‐only, regime‐only, abundance‐only, and persistence alternatives, while also preserving microbial attribution of methane and hydrocarbon dynamics.

**TABLE 5:** Day–1600 forecast ablation comparison for hydrocarbon concentration and methane production in ALBIAN and CNRL microcosms.

Model	ALBIAN				CNRL			
	Hydrocarbon		Methane		Hydrocarbon		Methane	
	Pred.	Error (%)	Pred.	Error (%)	Pred.	Error (%)	Pred.	Error (%)
Our model	540.20	37.91	2.49	4.42	1293.08	0.08	3.09	2.95
Full Shapley linear regression	541.54	37.75	2.54	6.55	1275.34	1.29	3.38	12.66
Abundance‐only Lasso benchmark	484.79	44.28	2.51	5.47	1295.15	0.24	3.06	1.85
State‐only Lasso ablation	306.60	64.76	2.38	0.03	1456.65	12.74	2.59	13.78
Regime‐only Lasso ablation	72.93	91.62	2.30	3.48	1419.33	9.86	2.82	6.10
Time‐only linear regression	822.17	5.50	3.06	28.69	1447.58	12.04	2.84	5.18
Logistic/saturation regression	959.10	10.24	2.20	7.56	1622.57	25.59	2.07	31.00
Day–1300 persistence benchmark	1042.00	19.77	2.20	7.56	1663.00	28.72	2.07	31.00

*Note:* The table reports predicted Day–1600 values and relative error (%) for each benchmark. True Day–1600 values are 870 mg/L hydrocarbon and 2.38 mmol methane for ALBIAN, and 1292 mg/L hydrocarbon and 3.00 mmol methane for CNRL.

Figure 8A,B presents a comparison of Shapley‐based hydrocarbon and methane reaction intensities for CNRL and ALBIAN across initial and late‐stage regimes. Hydrocarbon intensities are nearly identical for both sites, with slightly stronger degradation occurring in the initial stage. Methane intensities exhibit divergence: ALBIAN shows high levels initially, followed by a decline, whereas CNRL presents low levels at the outset, with a significant increase in the later phase. Comparative analysis of Shapley intensity components highlights that both degrade naphtha to a similar extent, with CNRL exhibiting delayed methanogenesis and ALBIAN demonstrating early but subsequently limited methanogenesis. This evidence indicates that a significant amount of naphtha does not necessarily correlate with rapid methane production; instead, the specific interactions among bacterial and archaeal communities, along with their early and late response dynamics, determine the rate of methane production.

**FIGURE 8:**
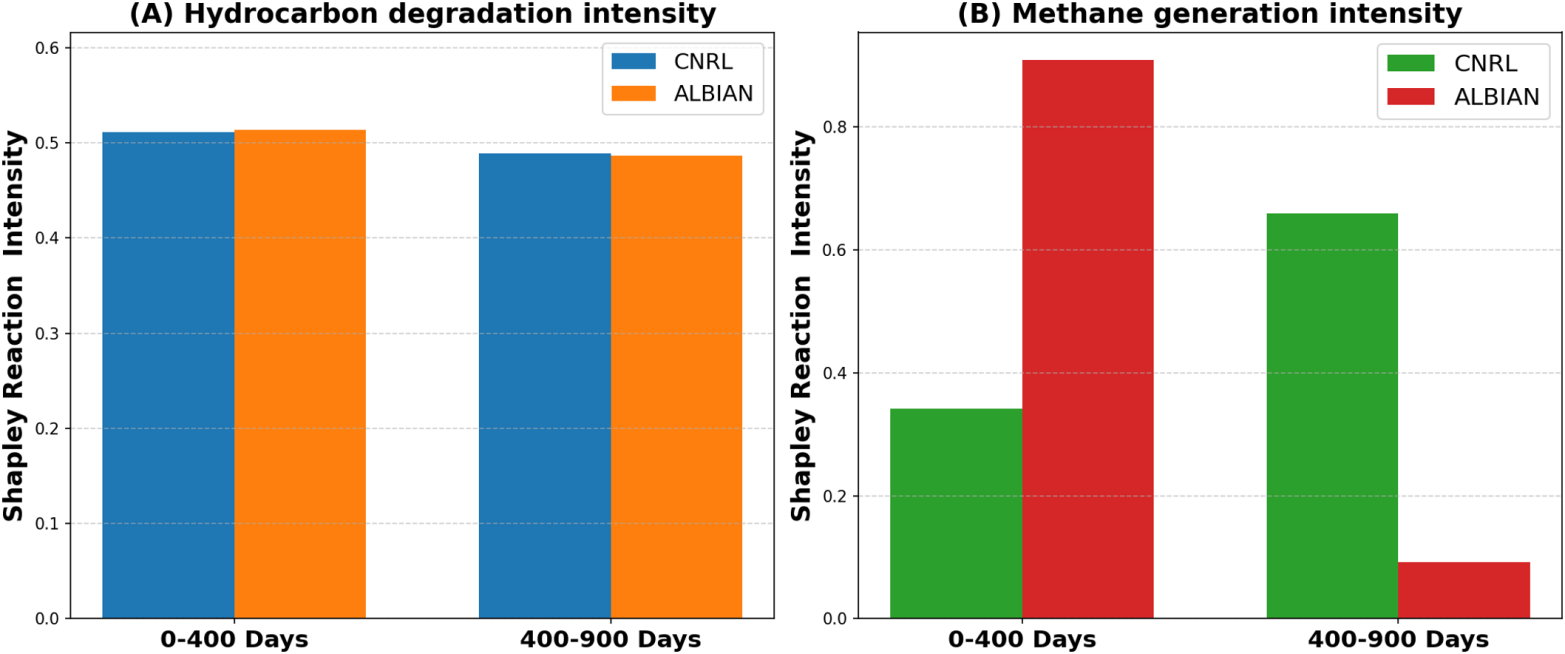
The figure illustrates reaction intensities for hydrocarbon consumption (left panel) and methane production (right panel) in microbial communities from ALBIAN and CNRL MFTs across two time regimes (0–400 days and 400–900 days). The results highlight a decoupling between hydrocarbon consumption and methane production, and the dominance of site‐specific metabolic regimes.

## 4 Discussion

The numerical results are consistent with the staged biodegradation behaviour reported for long‐term naphtha‐amended tailings incubations, but they also provide a coalition‐resolved interpretation of abundance‐informed community changes. The Shapley framework assigns interaction‐level contributions to hydrocarbon consumption and methane production, allowing dominant bacterial–archaeal partnerships to be identified within each incubation regime. Across both the uniform and non‐uniform allocation strategies, the pairwise Shapley analysis reveals consistent ecological patterns. In ALBIAN, the Clostridia–Methanoregula coalition contributes strongly to methane production during the initial phase, whereas in CNRL the same coalition becomes more influential at later stages. A related contrast is observed in CNRL, where Clostridia and Deltaproteobacteria show comparable contributions to hydrocarbon consumption, while coalitions involving Clostridia are associated with substantially greater methane production. This indicates that similar levels of hydrocarbon turnover do not necessarily correspond to similar methane outcomes, reflecting differences in how microbial partnerships contribute to the coupling between hydrocarbon degradation and methanogenesis. Importantly, the two alternative interpretations of the Shapley allocation yield the same dominant coalitions and temporal shifts despite differences in the magnitudes of individual pairwise contributions, providing a robustness check on the ecological conclusions. Thus, the framework quantitatively identifies the bacteria–archaea partnerships most strongly associated with methane‐forming processes in anaerobic tailings.

Recent studies have also identified hydrocarbon‐degrading archaea and alkylotrophic methanogens, including *Ca*. Methanoliparum, as important contributors to hydrocarbon‐associated methane production (Zhou, Zhang, et al. 2022). These findings show that methane production may not always depend only on classical syntrophic bacteria–archaea partnerships because some archaeal lineages can link hydrocarbon activation and methanogenesis more directly. However, *Ca*. Methanoliparum and related alkylotrophic methanogens were not resolved or reported as separate taxa in the 16S rRNA gene dataset used in this case study. The source dataset also does not include metagenomic, functional‐gene, isotope, metabolite, or expression data that would allow these archaea to be assigned distinct hydrocarbon‐consumption and methane‐production roles. Therefore, they are not included as separate players in the present implementation, although future applications of the framework can include such archaea directly when they are resolved in the available microbial and functional data. These distinctions matter for mitigation studies because strategies based on redox control or targeted amendments need to disrupt the interactions and functional pathways that sustain methanogenesis, not simply the taxa that appear most abundant (Afzal, Venegas Garcia, et al. 2025; Kuznetsova et al. 2023; You et al. 2020).

The case studies show the value of integrating relative‐abundance data with our Shapley‐based interpretations when forecasting or managing methane production in anaerobic tailings. In both ALBIAN and CNRL, several groups receive model‐implied methane contributions that differ from their relative‐abundance ranking (Table 3). The examples of Anaerolineae and Methanosaetaceae in ALBIAN, and Anaerolineae and other bacterial groups in CNRL, illustrate this mismatch clearly. The sensitivity analysis in Figure 2 shows that the principal methane‐attribution patterns remain consistent across alternative abundance‐weighted coefficient formulations. Because the ecological coefficients are constructed from relative‐abundance changes, the resulting Shapley attributions are not independent of abundance. They should therefore be interpreted as abundance‐informed, model‐implied contributions rather than direct measurements of microbial function. The λ‐sensitivity analysis evaluates robustness to alternative abundance‐weighted coefficient constructions, but does not remove this dependence on relative‐abundance data. This interaction‐based attribution enables comparison of microbial groups and cross‐domain partnerships across ecological regimes, providing quantitative pairwise attribution alongside conventional association‐based approaches (Yu et al. 2024; Siddique et al. 2020). For anaerobic tailings, where methanogenesis depends on distributed metabolic hand‐offs, this distinction is essential.

The forecasting results further indicate that hydrocarbon depletion and methane production remain temporally decoupled in naphtha‐amended tailings. CNRL shows slower mean hydrocarbon depletion, reaching an estimated endpoint near 2800 days, whereas ALBIAN reaches a comparable stage near 2500 days (Figure 7A,C). At the same time, methane production rises in both systems, with a steeper trajectory in CNRL and a higher mean value of 4.55 mmol compared with 3.10 mmol in ALBIAN (Figure 7B–D). The regime‐specific reaction intensities provide a mechanistic explanation for this site contrast: CNRL retains a stronger late‐stage methanogenic reaction intensity, whereas ALBIAN shows a weaker late‐stage response (Figure 8). This pattern is consistent with sequential depletion of more labile n‐alkanes in ALBIAN, followed by slower downstream fermentation and carbon transfer (Balaberda et al. 2026; Cossey et al. 2024; Zhang et al. 2024). The forecasting accuracy at 1600 days for both sites (Table 4) also indicates a clear improvement over theoretical methane yield estimates that do not account for regime‐dependent microbial interactions (Shahimin and Siddique 2017). The ablation benchmarks substantiate this interpretation by demonstrating that the Shapley‐based forecast yields the most robust coupled hydrocarbon‐methane performance in CNRL and surpasses the abundance‐only, state‐only, and regime‐only microbial benchmarks in ALBIAN, while preserving microbial interaction attribution that is not represented by trend‐only and persistence models (Table 5).

## 5 Conclusions

This study introduces a cooperative game‐theoretical framework that translates microbial community structure in anaerobic oil sands tailings into marginal and coalition‐aware contributions to coupled hydrocarbon transformation and methane generation. By distinguishing groups with larger model‐implied contributions from those with high relative abundance, the framework identifies bacterial–archaeal partnerships associated with disproportionate contributions to carbon transfer across incubation phases. This coalition‐resolved view provides a quantitative and reproducible link between shifts in community structure and changes in coupled hydrocarbon turnover and methane generation.

These findings are relevant for tailings management because they suggest that methane production is better interpreted through abundance‐informed microbial coalition structure than through taxon dominance alone. By identifying coalitions with the largest model‐implied contributions to hydrocarbon‐derived methane formation, the framework can help prioritize candidate microbial interactions for further investigation and potential management intervention. Such information could support the design of redox manipulation, amendment strategies, or other interventions, while recognizing that the inferred coalitions represent model‐based attributions rather than direct measurements of metabolic flux. More broadly, combining cooperative game‐based attribution with time‐series modelling and stochastic simulation provides a quantitative link between microbial community dynamics and predictive decision support for long‐term management of hydrocarbon‐impacted tailings systems.

Several limitations should also be recognized. The framework depends on sufficiently resolved time‐series measurements of microbial communities and coupled methane–hydrocarbon trajectories, and sparse or irregular sampling may reduce attribution precision. The ecological coefficients in this study are derived from changes in 16S rRNA gene relative abundance. Because absolute microbial loads, biomass estimates, or qPCR‐normalized abundances were not available, the analysis cannot distinguish whether relative‐abundance changes reflect growth of a given group, decline of other groups, or differences in total microbial abundance between samples or sites. They should therefore be interpreted as abundance‐informed coefficients, not as direct measurements of microbial activity or reaction rates. The λ‐sensitivity analysis evaluates the robustness of the attribution patterns to alternative abundance‐weighted coefficient constructions, but does not remove their dependence on relative‐abundance data. Future studies using absolute abundance, gene expression, isotope tracing, metabolite data, or direct rate measurements could provide stronger independent validation. The current regime‐based formulation also simplifies transitions between ecological phases and may miss short‐lived interactions that still influence methane production. In addition, Shapley values quantify marginal contribution within a defined value function, but they do not fully capture all nonlinear feedbacks, substrate thresholds, or context‐dependent asymmetries that can emerge during long‐term anaerobic biodegradation. The present application is based on the controlled incubation dataset, and should therefore be viewed as a case‐study demonstration of the proposed framework rather than a complete validation across all oil sands tailings systems. Finally, broader testing with independent datasets, additional tailings types, and a wider range of geochemical conditions will be needed to assess robustness, transferability, and field‐scale applicability.

Future work should expand the framework across additional tailings sources, hydrocarbon mixtures, and operational conditions to test how coalition structure varies under realistic field heterogeneity. Methodological extensions that incorporate more ecological phases, time‐varying interaction strengths, and feedback between microbial activity and geochemical state should further improve both mechanistic realism and predictive skill. Although this framework is developed for anaerobic hydrocarbon‐degrading tailings, its coalition‐based logic is general and can be adapted to other metabolically interdependent microbial systems, including aerobic oil sands environments structured by oxygen gradients and sulfate‐rich sediments where methane turnover depends on tightly coupled microbial consortia. In that broader sense, the framework offers a transferable way to quantify the model‐implied contributions of microbial partnerships to environmentally relevant carbon transformation processes in contaminated systems.

## Author Contributions

**Ali Hamidoğlu:** conceptualization, investigation, funding acquisition, writing – original draft, writing – review and editing, visualization, validation, methodology, software, formal analysis, data curation. **Hao Wang:** conceptualization, validation, supervision, project administration, writing – original draft, writing – review and editing, funding acquisition.

## Funding

This work was supported by the Natural Sciences and Engineering Research Council of Canada (NSERC) via an NSERC Alliance Missions grant (ALLRP‐2022‐577242) on anthropogenic greenhouse gas research.

## Ethics Statement

This study did not involve human participants, vertebrate animals, or regulated invertebrates and therefore did not require ethical approval.

## Conflicts of Interest

The authors declare no conflicts of interest.

## Data Availability

The two case study datasets analysed in this study are publicly available in Shahimin & Siddique (2017). All simulations were performed in Python 3. The code used for data analysis, simulation, and figure generation is available from the corresponding author upon reasonable request.

## Appendix A Shapley Cooperative Game Overview

This section provides preliminaries in cooperative game theory, including game value function, coalition framework, core of the game and Shapley value (Shapley 1953). Let $N=\left\{1,2,\ldots ,n\right\}$ denote the finite set of players, and let $2^{N}$ be the set of all possible coalitions. A cooperative game with transferable utility is traditionally defined as a mapping.

$$v:2^{N}\to \mathbb{R},$$

where $v\left(X\right)$ represents the value generated by coalition X⊆N.

In this study, we consider a two‐dimensional (vector‐valued) cooperative game, in which the characteristic function assigns to each coalition a vector payoff capturing two distinct dimensions of value. Formally, the game is defined as follows:

$$v:2^{N}\to {\mathbb{R}}^{2},\;v\left(X\right)=\left(\alpha_{X},\beta_{X}\right),$$

where $\alpha_{X}$ and $\beta_{X}$ denote the coalition value along the first and second dimensions, respectively. The motivation for adopting a two‐dimensional payoff structure is to explicitly represent the dual functional role of each microbial group, by assigning separate identifiers to its hydrocarbon‐related payoff and its methane‐related payoff. This formulation allows the model to distinguish between heterogeneous metabolic contributions that would otherwise be conflated in a single scalar value.

Following the linearity property of the Shapley value, the allocation for each player in a vector‐valued cooperative game can be defined component‐wise. For each player i∈N, the two‐dimensional Shapley value is given by:

$$\phi_{i}\left(v\right)=\left(\phi_{i}^{\alpha },\phi_{i}^{\beta }\right),$$

where $\phi_{i}^{\alpha }$ and $\phi_{i}^{\beta }$ represent player i's marginal contributions to the first and second dimensions of the game, respectively.

Specifically, the Shapley value(Shapley 1953) components are computed as:

(A.1)
$$\phi_{i}^{j}=\sum_{X\subseteq N\backslash \left\{i\right\}}\frac{|X|!\left(n-|X|-1\right)!}{n!}\left(j_{X\cup \left\{i\right\}}-j_{X}\right),$$

where $j\in \left\{\alpha ,\beta \right\}$. This component‐wise construction preserves the fundamental axioms of the Shapley value, namely efficiency, symmetry, dummy player, and additivity, within each dimension (Shapley 1953). Let us consider an allocation in the two‐dimensional game:

$$P=\left(P_{1},\ldots ,P_{n}\right),\;P_{i}=\left(P_{i}^{\alpha },P_{i}^{\beta }\right)\in {\mathbb{R}}^{2},$$

assigned to each player i∈N. The *core* of a two‐dimensional cooperative game is defined as the set of all allocations P that satisfy component‐wise individual rationality, coalitional rationality, and efficiency. More precisely, we have

- Individual rationality: $P_{i}≽v\left(\left\{i\right\}\right)$ for all i∈N.
- Coalitional rationality: $\sum_{i\in S}P_{i}≽v\left(S\right)$ for all S⊆N.
- Efficiency: $\sum_{i\in N}P_{i}=v\left(N\right)$.

Here ≼ presents the relation for component‐wise inequality in ${\mathbb{R}}^{2}$.

An allocation belonging to the two‐dimensional core ensures stability of the grand coalition by guaranteeing that no coalition can improve its payoff vector in either dimension through deviation. In this sense, the core represents an equilibrium–consistent stability concept for cooperative games, generalized here to a multi‐attribute payoff setting while preserving its fundamental equilibrium interpretation.

When examining the relationship between the Shapley value and the core of a cooperative game, it is well known that the Shapley value exists uniquely for every coalition game, whereas the core may be empty and, when non‐empty, is generally not unique. These distinctions persist in vector‐valued cooperative games, including the two‐dimensional payoff framework considered in this study. In this context, we introduce the notion of convexity for a two‐dimensional coalition game. Definition A.1 Let N denote the set of players and let $2^{N}$ be the set of all coalitions. A two‐dimensional cooperative game with characteristic function $v:2^{N}\to {\mathbb{R}}^{2}$ is said to be convex if, for any two coalitions S,T⊆N, the following component‐wise inequality holds:

(A.2)
$$v\left(S\right)+v\left(T\right)≼v\left(S\cup T\right)+v\left(S\cap T\right),$$

where ≼ denotes component‐wise inequality in ${\mathbb{R}}^{2}$.

A stable and fair distribution in the game is provided if the two‐dimensional cooperative game is convex, meaning that the core is non‐empty and contains the Shapley value (Shapley 1953).

## Appendix B Theoretical Foundations and Proofs

This section provides the theoretical foundations of the cooperative game framework, including the main results and their proofs that support the methodological development in the manuscript. Theorem B.1
*The two dimensional cooperative game under the value function* v *defined in* (1) *is convex*.
Consider arbitrary two coalitions:

$$S=\left(X_{s_{1}},\cdots ,X_{s_{p}},Y_{r_{1}},\cdots ,Y_{r_{q}}\right)\;\text{and}\;T=\left(X_{t_{1}},\cdots ,X_{t_{\ell }},Y_{h_{1}},\cdots ,Y_{h_{f}}\right),$$

$$\begin{array}{l} L_{S}≔\left(X_{s_{1}},\cdots ,X_{s_{p}}\right),R_{S}≔\left(Y_{r_{1}},\cdots ,Y_{r_{q}}\right),\\ L_{T}≔\left(X_{t_{1}},\cdots ,X_{t_{\ell }}\right),R_{T}≔\left(Y_{h_{1}},\cdots ,Y_{h_{f}}\right). \end{array}$$

We first prove the case when the coalition sequences S and T are disjoint, that is, S∩T=∅. By using properties of (1) and knowing v(X,Y)≽0 for any pairwise coalitions, we obtain

which proves the case.

Now, we assume the coalition sequences S and T have non‐empty intersection, that is, $S\cap T=\left\{L_{C},R_{C}\right\}$ where $L_{C}=L_{S}\cap L_{T}$ and $R_{C}=R_{S}\cap R_{T}$. Due to positiveness of the game value function and $L_{C}\subset L_{T}$ and $R_{C}\subset R_{T}$, we obtain:

(B.1)
$$v\left(L_{S}\backslash L_{C},R_{C}\right)≼v\left(L_{S}\backslash L_{C},R_{T}\right)\;\text{and}\;v\left(L_{C},R_{S}\backslash R_{C}\right)≼v\left(L_{T},R_{S}\backslash R_{C}\right),$$

where $L_{S}\backslash L_{C}$ and $R_{S}\backslash R_{C}$ simply represent set differences.

Using (Equation B.1) and the fact that $S\cup T=\left(L_{S}\backslash L_{C},L_{T},R_{S}\backslash R_{C},R_{T}\right)$, we obtain the following

Hence, we conclude that for any arbitrary coalitions S,T satisfy the convexity criteria (Equation A.2) which proves the theorem ■. Theorem B.2
*The Shapley values of the coalition game under the value function defined in (1) are given as follows*:

(B.2)
$$\phi_{X_{i}}=\left(\frac{M}{2}\sum_{\ell =1}^{k}\mu_{i\ell },\frac{C}{2}\sum_{\ell =1}^{k}\eta_{i\ell }+C\frac{\gamma_{i}}{k+1}\right),$$

(B.3)
$$\phi_{Y_{j}}=\left(\frac{M}{2}\sum_{\ell =1}^{m}\mu_{\ell j},\frac{C}{2}\sum_{\ell =1}^{m}\eta_{\ell j}-C\frac{\sum_{i=1}^{m}\gamma_{i}}{k\left(k+1\right)}\right),$$

*where* $i\in \left\{1,2,\cdots ,m\right\}$ *and* $j\in \left\{1,2,\cdots ,k\right\}$.
Since the value function is additive, namely:

$$v\left(X_{1},X_{2},\cdots ,X_{m},Y_{1},Y_{2},\cdots ,Y_{k}\right)=\sum_{i=1}^{m}v\left(X_{i},Y_{1},Y_{2},\cdots ,Y_{k}\right),$$

it is suffice to prove the case for $\left(X_{1},Y_{1},Y_{2},\cdots ,Y_{k}\right)$. Then, we use additive property to combine Shapley values of all $X_{1},X_{2},\cdots ,X_{m}$ for the general case. Hence, without loss of generality, we assume that m=1 and k≥1 arbitrary integer. Namely, we have k+1 total number of groups in the coalition game, represented as G (grand coalition). From the Formula (A.1), we provide the Shapley value of group player $X_{1}$ as follows:

From definition of the game value Function (1), any combination of $Y_{1},\cdots ,Y_{k}$, would provide zero coalition value. In addition, we know that:

(B.4)
$$v\left(X_{1},Y_{i_{1}},\cdots ,Y_{i_{j}}\right)=\sum_{\ell =1}^{j}v\left(X_{1},Y_{i_{\ell }}\right)=\sum_{\ell =1}^{j}\left(M\mu_{1i_{\ell }},C\eta_{1i_{\ell }}\right).$$

$$P_{1}≔\left(M\sum_{\ell =1}^{k}\mu_{1\ell },C\sum_{\ell =1}^{k}\eta_{1\ell }\right).$$

Using given information, we derive:

which proves the first part (Equation B.2) of the theorem. For the second part (Equation B.3) of the theorem, we derive the Shapley value explicitly for $Y_{1}$; the same argument applies analogously to each $Y_{j}$ for $j\in \left\{1,\cdots ,k\right\}$. Hence, we prove the case for $Y_{1}$, that is,

$$\phi_{Y_{1}}\left(v\right)=\left(\frac{M}{2}\mu_{11},\frac{C}{2}\eta_{11}-C\gamma_{1}\frac{1}{k\left(k+1\right)}\right)$$

Observe that for any $j\in \left\{1,\cdots ,k\right\}$

$$v\left(Y_{1},X_{1},Y_{2},\cdots ,Y_{j}\right)-v\left(X_{1},Y_{2},\cdots ,Y_{j}\right)=v\left(Y_{1},X_{1}\right),$$

which means marginal contribution of player $Y_{1}$ to any coalitions $\left(X_{1},Y_{2},\cdots ,Y_{j}\right)$ is fixed and equals to $v\left(Y_{1},X_{1}\right)$. Using that information and the Formula (A.1), we obtain:

which proves the second part (Equation B.3) of the theorem ■.
Theorem B.3
*The Shapley values given in Eqiations* (B.2) and (B.3) *constitute a stable and fair allocation of methane production and hydrocarbon consumption among the microbial groups within the anaerobic environment*.
Since the game under the value function (1) is convex according to Theorem B.1, marginal contributions of each microbial group are non‐decreasing as coalitions expand. Convexity indicates that the core of the game is nonempty and that the Shapley value lies within the core, thereby fulfilling the criteria of individual and coalitional rationality, in addition to efficiency. Therefore, no coalition of microbial groups can achieve a greater collective methane–hydrocarbon value by deviating from the grand coalition, resulting in the stability of the Shapley values. Fairness follows from the axiomatic characterization of the Shapley value. The allocation satisfies efficiency (the total value is fully distributed), symmetry (structurally identical groups receive equal shares), the dummy property (groups with no marginal contribution receive zero), and additivity. Therefore, under convexity, the Shapley values in Equations (B.2) and (B.3) provide a coalition‐stable and axiomatic fair distribution of marginal contributions within the anaerobic microbial system ■.
